# miRTrace reveals the organismal origins of microRNA sequencing data

**DOI:** 10.1186/s13059-018-1588-9

**Published:** 2018-12-04

**Authors:** Wenjing Kang, Yrin Eldfjell, Bastian Fromm, Xavier Estivill, Inna Biryukova, Marc R. Friedländer

**Affiliations:** 1 Science for Life Laboratory, Department of Molecular Biosciences, The Wenner-Gren Institute, Stockholm University, Stockholm, Sweden; 2 Genetics and Genomics Department, Sidra Medicine, Doha, Qatar

## Abstract

We present here miRTrace, the first algorithm to trace microRNA sequencing data back to their taxonomic origins. This is a challenge with profound implications for forensics, parasitology, food control, and research settings where cross-contamination can compromise results. miRTrace accurately (> 99%) assigns real and simulated data to 14 important animal and plant groups, sensitively detects parasitic infection in mammals, and discovers the primate origin of single cells. Applying our algorithm to over 700 public datasets, we find evidence that over 7% are cross-contaminated and present a novel solution to clean these computationally, even after sequencing has occurred. miRTrace is freely available at https://github.com/friedlanderlab/mirtrace.

**Electronic supplementary material:**

The online version of this article (10.1186/s13059-018-1588-9) contains supplementary material, which is available to authorized users.

## Introduction

An important laboratory challenge is to determine the organism or organisms from which a biological sample originates. In forensic science, clinical and field parasitology and food quality control, it is often critical to determine the identity of the animal or plant from which a sample is derived [1]. In research, next-generation sequencing allows transcriptomes to be profiled with unprecedented sensitivity, but this increases the risk that minute cross-species contamination compromises the results [2]. Commonly, ribosomal and mitochondrial DNA or other “barcoding” genes have been used to resolve the taxonomic origins of a sample [3, 4]. Specifically, short genetic markers that can be sequenced without species-specific PCR primers are used to identify a DNA sample as belonging to a particular species based on large databases of sequences for the particular locus. Importantly, the loci must therefore show high variation between species yet a relatively small amount of variation within a species. Accordingly, this method is sensitive to evolutionary sequence fluctuations such as back mutations [5, 6], and they require availability of references in the database.

MicroRNAs (miRNAs) are small RNAs that can regulate the expression of protein coding genes [7]. They are found in virtually all multicellular animals and plants [8], in numbers that approximately correspond to organismal complexity [9, 10]. Importantly, miRNA genes have emerged continuously over evolutionary time; they are rarely secondarily lost and are often highly conserved in sequence [11]. These properties give miRNAs potential as powerful phylogenetic and taxonomic markers. Indeed, miRNA genes have been successfully used to resolve many branches of the animal tree of life; a challenge related to, but distinct from, taxonomic tracing [11, 12]. However, when resolving phylogenetic branches, care must be taken when drawing conclusions from often incompletely annotated and incorrectly named miRNA complements [13, 14] as they can be misinterpreted as secondary losses [15]. Importantly, the evolutionary properties of miRNAs mean that each animal or plant clade (taxonomical group) has several miRNAs that are specific to that clade [16].

We have compiled known clade-specific miRNAs [11, 13, 17, 18] and developed an algorithm, *miRTrace*, that searches this catalog to reveal the taxonomical origin(s) of miRNA sequencing data. The currently preferred method to profile miRNAs is next-generation RNA sequencing, combined with specialized protocols that enrich for short transcripts. Our algorithm can be used directly for the quality control (QC) of such sequence data, and it can be adapted for more specific applications such as forensic science or parasitology. miRTrace stringently matches the sequenced miRNAs to the catalog of clade-specific miRNAs and reports on the overall composition. The method in this way identifies the taxonomical groups that contributed to the sample.

miRTrace has several potential advantages over the mitochondrial DNA-based barcoding gene approaches that are commonly used in animals and plants. First, since a given miRNA is either present or absent in a given clade, back mutations or sequencing errors cannot compromise the analysis. This is analogous to classical cladistics, which used morphological traits (present/absent) to resolve phylogenies [19]. Second, since miRNAs are conserved in sequence, our method works for completely unstudied species, provided that sequences from related species are present in our catalog. Third, since most clades have dozens of miRNAs that are specific to them, our method is robust to confounding factors that affect a single gene, such as secondary loss, duplication, or horizontal transfer.

We here give a proof-of-principle that miRNAs can be used to trace biological samples back to their taxonomical origins. Specifically, we show that miRTrace can track sequencing data back to 14 animal and plant clades with high (> 99%) accuracy. We also demonstrate the sensitivity of miRTrace, by detecting parasite-derived miRNAs in the blood of infected mice, and by discovering the primate origin of single human cells. A direct and important application of miRTrace is to detect cross-clade contamination in research sequencing data. We have analyzed more than 700 public miRNA sequencing datasets and find evidence that more than 7% have contaminations. Further, we identify index mis-assignment during sequencing as an important source of the contamination. We test common bioinformatics sequence analyses and find that they can be robust, even when 1% of the sequenced RNAs are cross-contaminations, and we present a new computational method to remove contaminations, even after sequencing has occurred.

Last, we have implemented miRTrace as fast and portable Java software. In addition to the taxonomical *Trace* functionality described above, our software performs an all-round *quality control* (QC) of miRNA sequencing data. These two functionalities or modes are described in two separate sections below. We validate the QC mode on in-house data from intentionally corrupted samples. The miRTrace software, including the trace feature, the quality control feature, and the contamination cleaning feature, is available at https://github.com/friedlanderlab/mirtrace.

## Results

### miRTrace: tracing miRNA sequencing data to their taxonomic origins

**Fig. 1:**
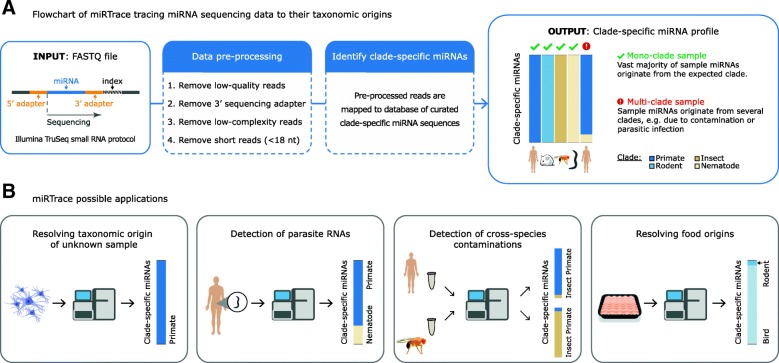
miRTrace: tracing miRNA sequencing data to their taxonomic origins. **a** Flowchart of how miRTrace assigns miRNA sequencing data to their taxonomical origins. One or more Fastq files and the synthetic adapter sequence are input. The data are pre-processed and clade-specific miRNAs are identified by perfect matching to a curated sequence database. The clade-specific miRNA profile is output in graphic format. **b** Possible miRTrace applications

The core function of miRTrace, to trace miRNA sequencing data back to their taxonomical origins, is performed in two steps (Fig. 1a). In the first step—the data pre-processing—low-quality sequencing reads are discarded, and the 3′ end sequencing adapter is removed from the remaining reads. This is important since when short miRNAs are sequenced, the sequencing reaction often extends into the adjacent adapter. Last, remaining reads that have low sequence complexity or that are shorter than 18 nucleotides are discarded (Methods). In the second step, clade-specific miRNAs are identified. Specifically, the remaining processed reads are mapped to our curated database of clade-specific miRNAs (Additional file 1), considering only perfect matches, and the composition of clade-specific miRNAs is reported as output (Methods). For each data set, the taxonomic composition is reported as a bar plot, with each of 14 different animal or plant clades presented with a distinct color (Fig. 1a, right). If a data set contains sequences from more than one clade, the bar will be stratified into distinct colors. This output can for instance be used to reveal the taxonomic origin of an unknown or complex sample, detect parasite RNAs or laboratory cross-species contaminations, or resolve the origins of food material (Fig. 1b).

### miRTrace accurately and sensitively traces sample taxonomic origins

**Fig. 2:**
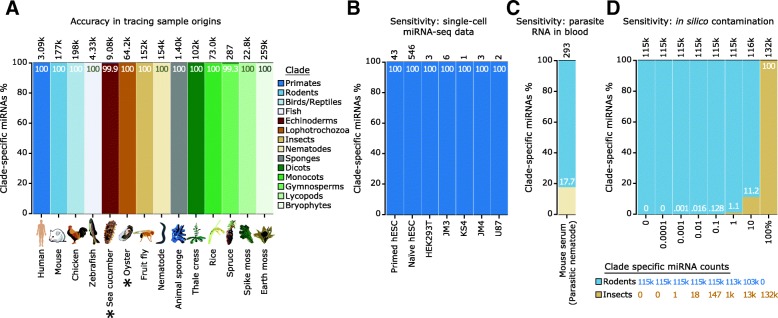
miRTrace determines the organismal origins of miRNA-seq data with high accuracy and sensitivity. **a** Representative miRNA-seq data from nine animal and five plant species were analyzed with miRTrace. The common species names are given below the bars. For each species, over 99% of the diagnostic clade-specific miRNAs were assigned to the expected clade, as shown by the bar color and the color key to the right. The total number of clade-specific sequencing reads in each dataset is shown above the bars. Sea cucumber and oyster (marked with black asterisks) were not represented in our reference database, but were still correctly identified as Echinoderm and Lophotrochozoan based on conserved sequences. **b** miRTrace identifies human samples as primate using data from single cells. **c** A parasitic infection is identified from nematode miRNAs in mouse serum. **d** Estimation of sensitivity. In a carefully controlled in silico experiment, fruit fly sequences were spiked into mouse data in abundances ranging from 0 to 100%. miRTrace detected the insect presence when 0.001% or more of the sequences were fruit fly

To test the accuracy of miRTrace in classifying unknown samples, we analyzed representative public miRNA sequencing data from 14 clades of animals and plants (Fig. 2a, Additional file 2: Figure S1; Additional file 3: Table S1; Additional file 4: Report S1). The method identified hundreds or thousands of clade-specific miRNAs in each species. In 12 species, 100% of these miRNAs belonged to the expected clade, and more than 99% of the sequences for the remaining species were assigned as expected. Importantly, the miRNA complement of two of the species, sea cucumber and oyster, has not been studied [20], and these species are not represented in our catalog. The method, however, correctly identified these species as echinoderms and lophotrochozoan, showing the generality and robustness of miRTrace.

We next tested the sensitivity of our method by analyzing miRNA sequencing data from single human cells [21]. Our method again correctly identified all clade-specific miRNAs as primate, showing that it can trace picogram sample material to the correct organism (Fig. 2b, Additional file 3: Table S2). We then used our tool to profile sequencing data from serum of mice infected with the parasitic nematode *Litomosoides sigmodontis* [22]. Both rodent and nematode miRNAs were detected (Fig. 2c, Additional file 3: Table S3), demonstrating that our method can detect parasitic infections in mammals. Next, we analyzed mouse sequences with different levels of fruit fly sequences introduced in silico (Fig. 2d, Additional file 3: Tables S4–S6). Fruit fly traces were detected when present at 0.001% or higher, showing that miRTrace can detect foreign miRNAs even when they comprise one in 100,000 sequences. Last, we applied binomial statistics to estimate the rate with which random sequence would match to our database of curated miRNAs (Additional file 2: Supplementary Methods). We found that a typical sequencing data set of ten million reads would produce less than one spurious match (Additional file 2: Figure S2), demonstrating that our method is highly specific.

### Evidence that cross-clade contamination is prevalent in public miRNA sequencing data

**Fig. 3:**
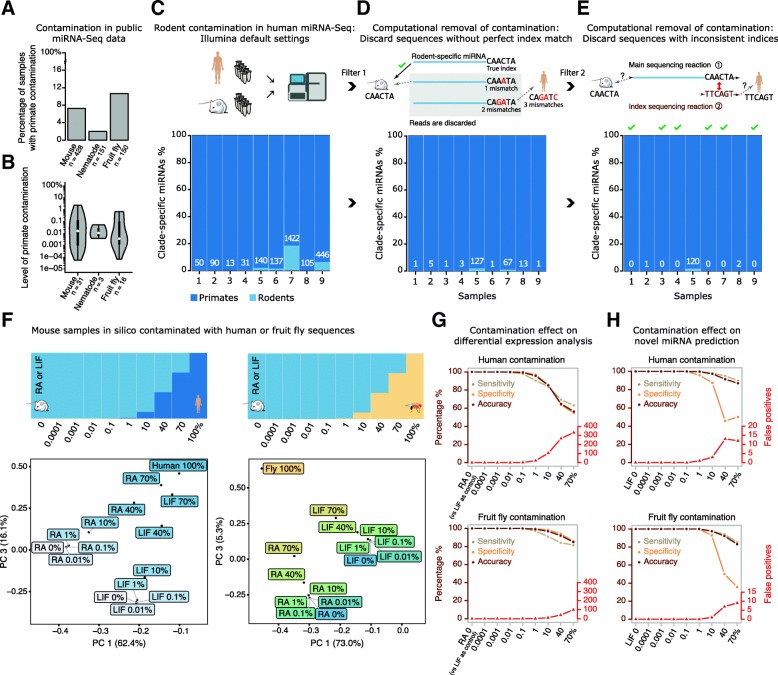
Prevalence, causes, and effects of cross-contamination in miRNA-seq data. **a** Percentages of public mouse, nematode, and fly datasets that contain primate sequences (putative human contamination). **b** Percentages of primate sequences in putatively contaminated datasets (considering only clade-specific sequences). The white dot indicates the median value, the thick black line indicates the range from 25 to 75% percentiles, and the whole gray area indicates the range from 5 to 95% percentiles. **c** Human and mouse samples were profiled in parallel with Illumina sequencing to detect sources of cross-contamination (top). Using the Illumina default settings for sample assignment, rodent contamination was detected in all nine human samples. Each bar represents one sample, and the numbers in each bar indicate the number of contaminating sequences (bars below). **d** Using more stringent settings for sample assignment, only sequences with perfect matches to known sample indices were retained (top). This computational step removed most rodent contaminations (bottom). **e** In an additional filtering step, sequences with inconsistent indices were discarded (top), removing contaminations completely from six out of nine human samples (bottom). **f** Mouse samples in silico contaminated with controlled abundances of human or fly sequences ranging from 0 to 100% (top). Principal component analyses (PCA) show how the overall miRNA composition of the sample changes with increasing levels of contamination (bottom). **g** Effects of the contaminations from (**f**) on gene differential expression (DE) analyses. Sensitivity, specificity, and accuracy (in shades of brown) are given as fractions, while the false positive rates (in red) are absolute numbers. **h** Effects of contamination on the prediction of novel miRNAs

In research settings, cross-species contamination of samples can reduce reproducibility and complicate the interpretation of results. One direct and important application of miRTrace is to detect such contamination in miRNA sequencing data. We surveyed more than 700 public research sequencing datasets from mouse, nematode, and fruit fly for the presence of primate miRNAs, since the most plausible explanation for the presence of primate sequences in mouse, nematode, or fly would be human contamination, from, for example, researchers or patient material (Fig. 3a, Additional file 2: Figure S3, Additional file 3: Tables S7–S12, Additional file 4: Reports S2–S4). On average, 7% of the datasets contained primate sequences, with more in the fruit fly data (11%) and less in the nematode data (2%). However, the levels of the putative contaminants were generally low, with a median contamination level from primate sequences of 0.01% of the clade-specific miRNAs (Fig. 3b). We conclude that trace levels of cross-contaminants are prevalent in public research sequence data.

### Index mis-assignment is an important source of contamination

We next designed a controlled in-house experiment to study potential sources of cross-contamination. Extensive research has been conducted on this topic for DNA sequencing [2, 23], but none specifically for miRNA sequencing. Work with DNA has shown that cross-contamination can occur during the sequencing process, and thus we used the Illumina NextSeq500 instrument to profile miRNAs from human cell cultures and mouse neuronal synapses in parallel. Mouse synapses contain highly abundant rodent-specific miRNAs, and we were therefore not surprised to observe clear rodent contamination in all of our nine sequenced human samples (Fig. 3c, see Additional file 2: Note S1 and Figure S4 for a discussion on the effect of miRNA annotation imbalances between species, Additional file 3: Table S13). When multiple samples are profiled in parallel, each detected miRNA is assigned back to its sample of origin based on an *index* sequence that is unique to that sample. If some of the nucleotides of the index are misread during the sequencing, the miRNA may be assigned to an incorrect sample (Fig. 3d, top, Additional file 2: Figure S5). We therefore repeated the assignment, but stringently discarded miRNAs with indices that did not have a perfect match to a known sample index. This step removed approximately 90% of the rodent sequences from the human samples, indicating that index misreading is an important source of miRNA contamination (Fig. 3d, bottom, Additional file 3: Table S14).

Cross-contamination can also occur when the sequencing instrument reads an index correctly but pairs it to an incorrect transcript (Fig. 3e, top). This is possible because the index and the transcript are read in two distinct sequencing reactions, which can then be mis-paired by the instrument [2]. However, since miRNAs are short transcripts, when they are sequenced, the main reaction can extend into the adjacent index. For instance, the default 75 bp Illumina TruSeq kit should be long enough to cover miRNA (~ 22 nt), adapter sequence (33 nt), and index (6 nt). A consequence of this is that the index is read twice. We developed software to identify and discard miRNAs with inconsistent indices and in this way remove contaminated samples that originate from mispairing events. We successfully used this approach to completely clean all contamination from six of nine samples (Fig. 3e, bottom, Additional file 3: Table S15). We compared our method of removing mis-paired indices with a previously described approach that filters sequencing reads based on index sequencing quality [24], and found that the two compare similarly in cleaning efficiency, while our method retains more useful miRNA sequences (Additional file 2: Figure S6). Further, our method works independently of index quality scores, which are rarely available for public data. In summary, our results show that misread and mis-paired indices are sources of miRNA cross-contamination, and we present a method to computationally remove them, after the samples have been sequenced. Importantly, our cleaning method can remove same-species contamination and can also be used for other short transcript applications such as CLIP-seq [25, 26].

### Impact of contamination on downstream bioinformatical analyses

Having studied the sources of cross-contamination, we turned to their effects on subsequent bioinformatical analyses. We computationally spiked in either human or fly miRNA sequences into data from mouse embryonic stem cells (Fig. 3f, top, Methods, Additional file 3: Tables S16, Additional file 4: Reports S5–S6). The mouse cells had been cultivated either in the presence of a compound that maintained their pluripotency (leukemia inhibitory factor, LIF) or with a compound that induced them to differentiate into neurons (retinoic acid, RA) [27]. Contaminants at levels of 1% or less did not change the overall transcriptional profile of the samples, as indicated by principal component analyses (PCA), while higher levels of contaminants increasingly changed the profile towards that of the contaminating sample (Fig. 3f, bottom, Additional file 2: Figures S7–S8, Additional file 3: Tables S17–S18). We next used differential gene expression analysis to identify genes that had been activated following induction into neurons (RA vs. LIF). The analysis was not compromised by the presence of up to 0.1% human sequences or 1% fly sequences, while higher levels of contaminants reduced the sensitivity and caused false positives (Fig. 3g, Additional file 2: Figures S9–S10, Additional file 3: Table S19). The analyses performed on mouse data were more sensitive to the human sequences than to the fly sequences, and we speculate that this is because of the closer evolutionary relationship. Finally, we predicted novel miRNA genes from the mouse LIF data using miRDeep2 [28] (Methods). The presence of up to 0.1% human or 1% fly sequences did not disturb the analyses, while a higher level of contaminants reduced sensitivity and caused some false positives (Fig. 3h, Additional file 3: Table S20). These results indicate that common analyses can be robust to levels of contaminants up to 1%, depending on species relatedness and the type of analysis. This leads to the conclusion that approximately 1.5% of the public datasets may be substantially compromised by contaminants (Fig. 3a, b).

### miRTrace: all-round quality control of miRNA sequencing data

We have implemented the algorithm described in this study as fast and portable Java software. It operates in two modes: *Trace* mode, in which the software reports the composition of clade-specific miRNAs of unknown or complex samples, as described above, and extended *QC* mode, in which it performs an all-round quality control of miRNA sequencing data, applicable to any of the 219 animal or plant species currently available in the miRBase database [20].

**Fig. 4:**
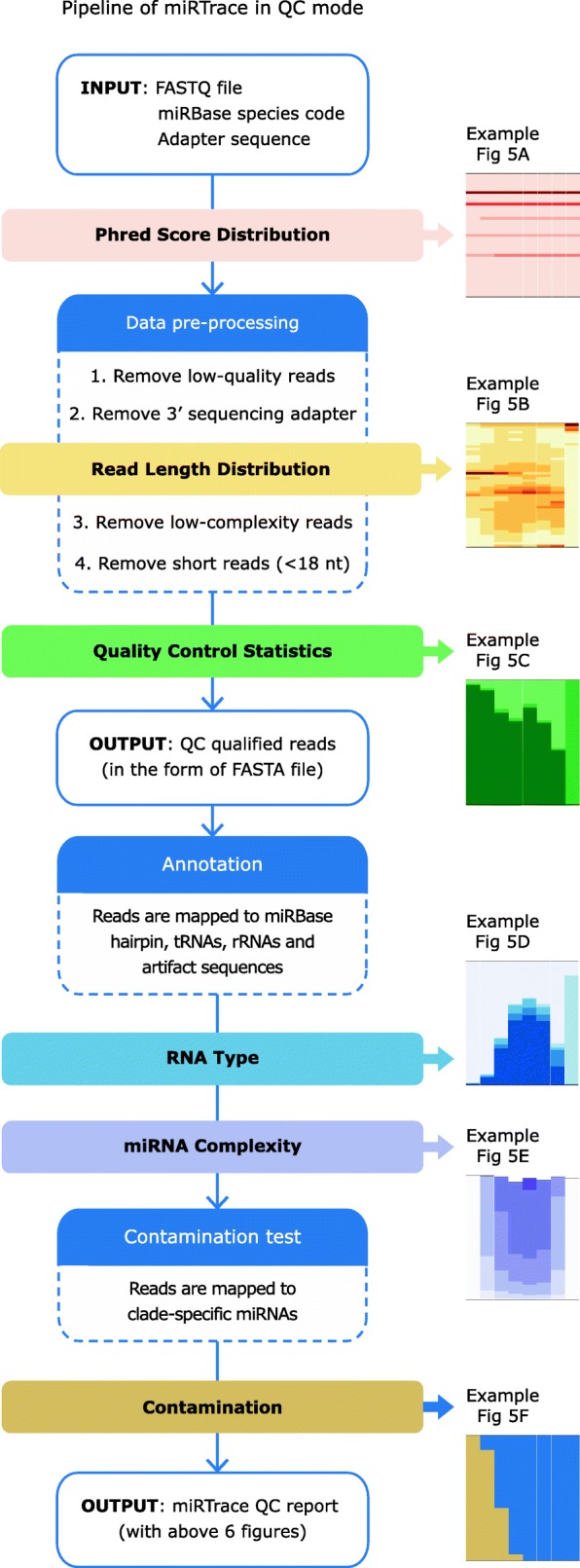
miRTrace: an all-round quality control tool for miRNA sequencing data. Flowchart of the steps that generate the six reports shown in Fig. 5a–f. For instance, an example of first report is shown in Fig. 5a. A detailed description of the flowchart can be found in the Results section

**Fig. 5:**
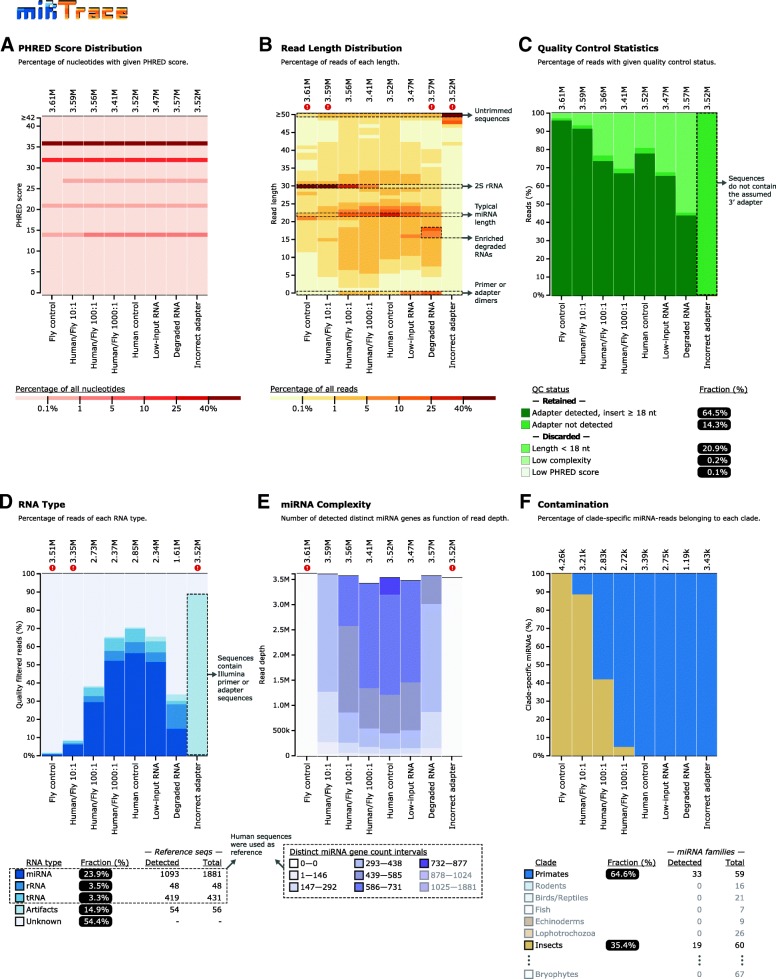
miRTrace quality control report. **a** Sequencing quality. Higher Phred scores indicate higher sequencing quality. In this case, all samples pass the filter. The number above each column shows the number of sequences in the dataset. **b** Length of sequenced transcripts. miRNAs are ~ 22 nucleotides long, while the fly 2S RNAs are ~ 30 nucleotides long. Zero-length sequences indicate adapter artifacts, while lengths of > 50 indicate that the sequencing adapter has not been successfully identified. Four samples are flagged because < 25% of the sequences are between 20 and 25 nucleotides long. **c** Quality control. After the ligation adapter has been removed, sequences are grouped according to length and sequencing quality. The most desirable sequences have the adapter removed and are long enough to map reliably (18 nucleotides or longer). **d** RNA type. For most applications, a high miRNA content and low rRNA and tRNA content are desirable. Illumina sequences are tagged as “artifacts.” Here, three samples are flagged because the miRNA content is < 10%. **e** miRNA sequence complexity. Each bar shows the number of distinct miRNA sequences as a function of sequencing depth. Two samples are flagged because < 10% of all known miRNAs for the studied species (human) is represented in the sample. **f** Contamination. The human samples that are intentionally contaminated with fly clearly stand out (in beige color). In total, 14 clades are considered, but here only eight are shown in the table below. Samples: Fly control, S2 cells; Human/Fly 10:1, total RNA from HEK-293T cells mixed with total RNA from S2 cells in ratio 10:1; Human control, HEK-293T cells; Low-input RNA, 50 ng total RNA from HEK-293T cells; Degraded RNA, HEK-293T total RNA incubated with RNase A enzyme; Incorrect adapter, wrong adapter sequence designated when starting the analysis. The six report figures here are imported directly from miRTrace output, except for the small gray arrows and the accompanying annotations, which have been added here for clarification

The *QC* mode builds on the workflow of the *Trace* mode (Fig. 1a), but more steps are included and in total six report figures are generated during the flow (Fig. 4). First, a report figure of the sequencing quality is output (“Phred Score Distribution,” example Fig. 5a). In the data pre-processing step, low-quality reads are discarded, the 3′ end sequencing adapters are removed, and low-complexity reads and short reads are discarded (Methods). After the adapters have been removed, a report figure is generated that shows the length profile of reads (“Read Length Distribution,” Figs. 4 and 5b). The length profile of sequenced transcripts can be highly informative, since particular classes of transcripts have distinct lengths, for instance, miRNAs are often 22 nucleotides long. Next, quality control statistics accumulated thus far are reported, showing what fractions of reads have been discarded or retained in the previous steps (“Quality Control Statistics”, Figs. 4 and 5c). In the annotation step, reads are mapped to a custom database of miRNA precursors, tRNAs, rRNAs, and synthetic adapter sequences that may be present, and the composition of each data set is presented (“RNA Type,” Figs. 4 and 5d). From the sequences that have now been identified as miRNAs, a report is generated showing the number of distinct miRNAs in each data set, as a function of sequencing depth (“miRNA Complexity,” Figs. 4 and 5e). Ideally, many distinct miRNAs should be present in each data set; low complexity data sets may be generated when low quantities of RNA are sequenced. Last, in the contamination test step, sequence reads are mapped against our catalog of clade-specific miRNAs, identical to the *Trace* mode (“Contamination,” Figs. 4 and 5f). This is a key part of the *QC* analysis, since cross-species miRNAs could result from contamination that compromises the data.

We have benchmarked the *QC* mode on a small use case representative of a research laboratory, and on an extensive use case representative of a bioinformatics or sequencing facility. The analysis of ten datasets comprising approximately 185 million reads takes less than 8 min on a MacBook Air (2014) laptop with 2 GB memory allocated, while the analysis of 428 datasets comprising approximately 4.5 billion reads takes less than 14 min on a server with 20 cores (Xeon(R) E5-2630 v4) and 32 GB memory allocated, demonstrating the efficiency of our software.

### miRTrace quality control accurately identifies problematic samples

To test our software on realistic data, we have validated the *QC* mode using in-house human RNA control samples that we intentionally subjected to various insults. These treatments include cross-species contamination, sample dilution, and RNase A digestion (Fig. 5, Methods, Additional file 3: Table S21). As expected, miRTrace successfully identifies fruit fly contamination in human samples even when it is present in a 1:1000 ratio at the RNA level (first four bars in Fig. 5f). In addition, miRTrace also discerns subtle changes in quality caused by contamination. For example, the human miRNA content and sequence complexity decrease as the fly presence increases (Fig. 5d, e). The changes are also reflected in the overall sequence length profile, since the abundant fly 2S RNA is around 30 nucleotides long, while the human miRNAs are around 22 nucleotides long (Fig. 5b).

miRTrace also discerns the differences between the human control sample and a sample that has been partly degraded with RNase A enzyme. The degraded sample has fewer sequences that are around 22 nucleotides long, and a higher fraction of shorter variants, including zero-length sequences, which are probably ligation adapter artifacts (Fig. 5b). The degraded sample also has a lower miRNA content and sequence complexity (Fig. 5d, e). These tendencies are observed to a lesser extent for the low-input sample that was prepared from only 50 ng total RNA. Last, when an incorrect ligation adapter sequence is provided to miRTrace, no adapters are identified (Fig. 5c) and more than 85% of reads are labeled as “Artifacts,” which suggests that the unclipped adapter sequences are aligned to our Illumina artifact database (Fig. 5d). In summary, miRTrace accurately identifies poor-quality samples and can even, to some extent, provide hints to the cause of the poor quality.

### The miRTrace method can trace samples to the species level

**Fig. 6:**
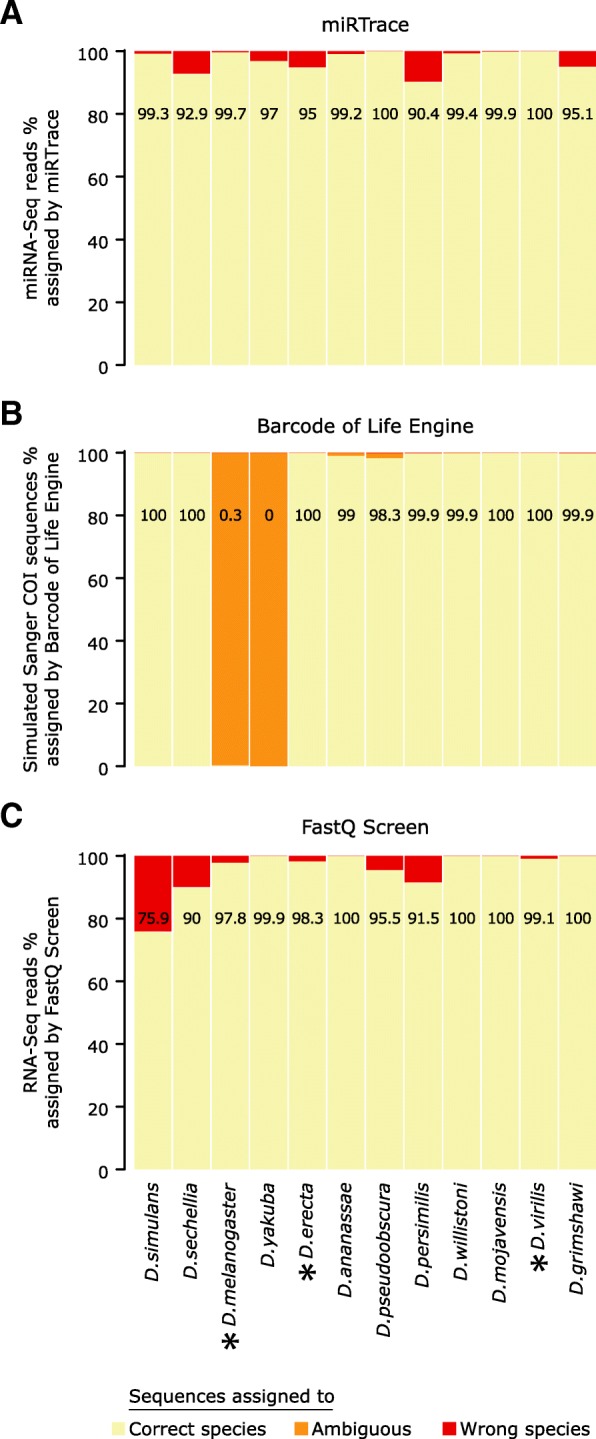
miRTrace can be refined to trace samples to the species level. **a** miRNAs known to be specific to individual *Drosophila* species were compiled to generate a custom database for miRTrace. miRNA sequencing data from the 12 species were obtained from public repositories and assigned to the species by miRTrace, using the custom annotations. The number in each bar indicates the percentage of sequences that were traced to the correct species (pale yellow). **b** Sanger sequences of the COI gene were simulated for the 12 *Drosophila* species, considering intra-species variation and sequencing error rates, and assigned using the Barcode of Life Identification Engine. For each species, 1000 sequences were simulated. **c** RNA sequencing data from the 12 *Drosophila* species were obtained from public repositories and assigned using the FastQ Screen tool. The black asterisks indicate species where it was possible to obtain matched miRNA and RNA sequencing data (obtained from the same study)

While some studies have provided miRNAs that are specific to broader clades [11, 17, 18], currently few truly species-specific miRNAs are known. However, a recent study has provided a catalog of miRNA candidates specific to each of 12 *Drosophila* species [29]. This has allowed us to evaluate if our method can trace miRNA sequencing data to the species level, when the required annotations are available. Specifically, we analyzed public miRNA sequencing data from the 12 *Drosophila* species, while providing curated species-specific databases to miRTrace. For all 12 datasets, > 90% of the reads were correctly assigned and the species of origin was unambiguously identified (Fig. 6a, Additional file 3: Table S22), demonstrating that our approach can accurately trace to the species level, when the annotations are available.

This case study also allowed us to compare miRTrace to other methods, at the species level. Currently, miRTrace is the only approach that can trace miRNA sequencing data to their taxonomical origins, but similar methods exist for other data types. The most common method for taxonomical tracing in animals uses barcoding of the mitochondrial Cytochrome Oxidase C Subunit 1 (COI) gene [4]. This method does not analyze next-generation sequencing data, rather researchers extract DNA from samples of interest, PCR amplify the COI gene, and sequence it with Sanger sequencing, before mapping it to a comprehensive COI sequence database [4]. We therefore simulated Sanger sequencing data from the 12 *Drosophila* species, considering intra-species sequence variation and common error rates of Sanger sequencing (Additional file 2: Supplementary Methods). We found that the Barcode of Life Engine assigned more sequences correctly than miRTrace for seven of the 12 species, while miRTrace performed better for four species, and both methods performed perfectly on one species (Fig. 6a–b) However, for two of the species, most Sanger sequences could not be correctly assigned to a single species. When we looked closely into these species, we found that the ambiguous assignment is likely due to incorrectly annotated sequences in the Barcode of Life reference database, and indeed when comparing all available *Drosophila* COI sequences, it was not possible to assign all of the species to monophyletic groups (Additional file 2: Figure S11).

While currently no competing methods exist to trace miRNA sequencing data to their origins, there are similar methods for other next-generation sequencing data. The FastQ Screen tool maps long reads produced by RNA or DNA sequencing simultaneously to multiple genomes of interest, and reports how many reads map unambiguously to the expected genome, and how many reads map to other genomes [30]. When mapping publicly available RNA sequencing data with FastQ Screen, we found that reads were assigned to most *Drosophila* species with > 90% accuracy, similar to the performance of miRTrace (Fig. 6c, Additional file 2: Supplementary Methods, Additional file 3: Table S23). miRTrace outperformed FastQ Screen for five out of 12 species, while FastQ Screen performed better for seven species (Fig. 6a, c). Importantly, for three of the species (*D. melanogaster*, *D. erecta* and *D. virilis*), we found RNA sequencing data that matched the miRNA sequencing data analyzed by miRTrace above (exact same studies); miRTrace performed best for two out of three of these species. This test case is likely particularly challenging for FastQ Screen, since some of the *Drosophila* species are closely related, and several have incomplete genome assemblies. In summary, we find that miRTrace performs comparably to state-of-the-art tools that trace other types of sequence data to their taxonomic sources. However, the types of mis-assignments (ambiguous or wrong assignments) differ between the tools, suggesting that the approaches may be complementary.

## Discussion

We have here given a general proof-of-principle that miRNAs can be used to trace biological samples back to their taxonomic origins. This approach has several advantages over traditional methods—in particular, that the analysis is binary and conceptually simple, since a given miRNA is either present or absent from an animal or plant clade. We also present miRTrace, an implementation of the method that accurately and sensitively traces miRNA next-generation sequencing data to 14 important animal and plant clades. A primary application of miRTrace is to detect contaminations in miRNA research data. We have surveyed more than 700 public datasets and have presented evidence that more than 7% are compromised. We have identified index mis-assignments during sequencing as an important source of contamination, and we have proposed a simple yet innovative method to clean contamination, even after sequencing has occurred. Importantly, miRTrace is available as general-purpose quality control software and is fast and user-friendly, making it useful for bioinformatics and sequencing facilities and for the individual biologist.

We have shown that miRTrace is quantitative for some clade comparisons (Fig. 2d), while it reports skewed relative abundances for other comparisons (Fig. 5f). This has biological reasons, since some clades have larger sets of miRNA genes that are specific to them and that are more abundantly expressed (see the Additional file 2: Note S1 and Figure S4). This could easily be corrected by profiling miRNA genes at the DNA level, similar to the methods normally used to analyze barcoding genes. However, there are several advantages to profiling miRNAs at the RNA level. First, the RNA molecules are often present in thousands of copies per cell, compared for instance to the two autosomal DNA copies in diploid cells, making the profiling more sensitive. Second, there have long existed protocols to separate the total pool of miRNAs from other transcripts [31–33], without the need for specific primers or probes. Third, miRNAs are very stable transcripts [34], detectable even in minute dried blood samples [35] or from samples several thousands of years old [36]. All these features combined make miRNAs very promising markers at the RNA level.

Similar to the advantage of having thousands of miRNA copies per cell, there are advantages to using multiple gene loci over single gene loci for taxonomical tracing. During evolution, individual genes can be lost or sequence can revert back to ancestral forms. Further, sequencing errors can obfuscate differences between closely related species, and mis-annotation in databases can make it difficult to assign particular genes unambiguously. By leveraging on multiple genes for tracing, the impact of events that affect one gene can be minimized, and the analyses can be made more robust.

Previous studies and our analyses here show that long reads produced by RNA sequencing can also be traced to the species level, using FastQ Screen [30] (Fig. 6c). However, since this method relies on mapping to whole genomes, the sequence space that needs to be searched is in the range of billions of nucleotides, which makes the tracing computationally demanding. Our entire curated miRNA database, covering 14 important animal and plant general clades, comprises around 30,000 nucleotides, making the search computationally efficient and also highly specific, with less than one expected false positive for every ten million reads (Additional file 2: Figure S2). This makes miRNAs particularly useful for applications where high-performance computing may not be available, such as in-the-field tracing analyses.

Current catalogs of clade-specific miRNAs are far from complete [13, 20]. With saturated annotations, miRTrace could give complete coverage from phylum to species, identifying marker miRNAs along entire branches of the phylogenetic tree. This would simultaneously yield both high-level and fine-resolution taxonomic information in a single analysis, for increased robustness. In the *Drosophila* test case (Fig. 6), we have shown that such a detailed tracing is possible both in theory and in practice when species-specific annotations are available.

Finally, single-celled eukaryotes, prokaryotes, and viruses also have small RNAs [37–39]. If these were to be included in our catalogs, our approach could be extended to all the domains of life, making it a strong complement to the traditional barcoding methods. Thus, we propose that a future initiative to complete the annotations of small RNAs, similar to the existing Barcode of Life Project [40], would benefit numerous research fields.

## Conclusions

We conclude that miRNAs can be used to trace biological samples back to the clade or even species of origin. We have shown that miRTrace can accurately (> 99%) assign miRNA sequencing data to 14 animal and plant clades, sensitively determine the taxonomic origins of single cells, and detect parasitic nematode miRNAs in mammalian host blood samples. We estimate that > 7% of public miRNA sequencing datasets may be cross-clade contaminated and that common bioinformatics analyses are robust to up to 1% contamination. We find that index mis-assignment during sequencing is an important source of cross-contamination, which can in some cases be cleaned computationally after sequencing. miRTrace is available as fast and portable software—to trace, quality control, and clean miRNA sequencing data—and importantly, its architecture allows for future expansion as new miRNA reference data become available.

## Methods

### miRTrace pipeline

miRTrace is a portable JAVA program for quality control (QC) and tracing taxonomic origins of small RNA sequencing data. miRTrace takes FASTQ files as input. If the reads are longer than 50 nucleotides (nt), the sequence beyond the first 50 nt is trimmed. Low-quality reads with more than 50% nt with PHRED score less than 20 are removed. The remaining reads are subjected to adapter trimming. For the Illumina TruSeq small RNA and the QiaSeq miRNA protocols, the reads are searched for the first 8-mer of the 3′ adapter. If a match is found, the last appearing match is removed, together with all subsequent nucleotides. If no match is found, miRTrace then scans and trims the 3′ end of reads that have the largest match to the 5′ end of the 3′ adapter. For the NEXTflex Small RNA-Seq protocol, first the four initial nt are trimmed and the 3′ adapter sequence is removed as described above. Additionally, if the adapter is detected, the last four nt are trimmed. For the CATS Small RNA-seq protocol, the three initial nt are trimmed and the left-most occurrence of a poly-A 8-mer is searched for. If a match is found, the poly-A tail and subsequent nt are removed. Otherwise, miRTrace attempts to match and trim using subsequently shorter poly-A k-mers (down to a single A if necessary). After adapter removal, the reads containing any ambiguous nt (e.g., N), the reads containing highly repeated nucleotides, and the short reads (< 18 nt) are removed. The adapter trimmed reads with length ≥ 18 nt are named as “QC-passed reads,” as are also adapter unrecognized reads (sequences where no adapter has been trimmed), and used for later analysis.

To know the RNA composition of a sample, each QC-passed read is first aligned to miRNA precursor sequences downloaded from miRBase v21 [41], then tRNA sequences downloaded from tRNAdb and mitotRNAdb [42] (http://trnadb.bioinf.uni-leipzig.de), and then ribosomal RNA sequences curated from NCBI nucleotide, Silva [43] (https://www.arb-silva.de) and the Ensembl database, without allowing any mismatches. If no alignment is found, miRTrace will redo the mapping with one mismatch allowed. The reads are annotated based on the type of RNA that they first aligned to. Furthermore, the remaining unmapped reads are aligned to the “artifact sequences” from Illumina adapter documents, requiring at least one 18 nt stretch of sequence identity. The reads that do not map to any of the above databases are marked as “unknown.” All of the reference databases can be found in the “reference_databases” folder of the miRTrace package.

To estimate the miRNA sequence complexity, miRTrace calculates the cumulative number of distinct miRNA precursors identified in a given dataset as a function of sequencing depth. The number of distinct miRNA genes is determined by the number of distinct precursor sequences that the reads map to, while the sequencing depth is determined by the number of processed raw reads. A sample with high miRNA complexity tends to have a greater number of distinct miRNA precursors and to reveal more lowly abundant miRNAs as sequencing becomes progressively deep.

According to the previously curated clade-specific miRNA family numbers (Additional file 1), the reference catalog of clade-specific miRNA sequences is obtained from miRBase v21 by selecting the mature miRNA sequences with an ID that contains the family numbers. For example, the primate-specific miRNA family 580 yields five sequences with miRBase v21 IDs hsa-miR-580-5p, hsa-miR-580-3p, mml-miR-580, ptr-miR-580, and ppy-miR-580. A read is identified as clade-specific miRNA if its first 20 nt have an exact match to a reference sequence. By counting the number of clade-specific miRNAs of each clade and the number of unique clade-specific miRNA sequences, miRTrace provides the clade and sequence count table of clade-specific miRNAs respectively.

### Public small RNA-Seq data pre-processing

More than 700 public sRNA-Seq datasets were used for the study. The datasets were downloaded from the NCBI SRA database (https://www.ncbi.nlm.nih.gov/sra) and detailed information of these samples can be found in Additional file 3. The datasets in SRA format were converted to FASTQ files using fastq-dump from SRA Toolkit v 2.3.5. The 3′ adapter of each sample was identified individually using the same approach as in Kang et al. 2017 [44] (see “Methods” section “De novo 3’ adapter identification”). Notably, the single-cell libraries (*n* = 7) from Faridani et al. 2016 [21] have custom designed 5′ adapters with the unique molecular identifier sequence (UMI) at the 3′ end, which makes the reads un-processable by miRTrace. The extra UMI sequences (the first 10 nt of the reads) of these samples were for this reason trimmed. All FASTQ files were then processed by miRTrace to profile clade-specific miRNAs.

### Culturing human and fruit fly cell lines

HEK-293T cells were cultured in DMEM (Sigma-Aldrich) supplemented with 10% FBS (Gibco); *Drosophila S2* cells (Invitrogen) were cultured in Schneider cell medium (Gibco) with 10% FBS under standard conditions.

### Degradation by Ribonuclease A

To perform enzymatic degradation of RNA samples, a partial hydrolysis by Ribonuclease A was used. Ten micrograms of HEK-293T total RNA samples was hydrolyzed by 10 ng of RNAse A (Thermo Fisher) at room temperature for 5 min. The activity of RNAse A was inhibited by RNAseOUT (Invitrogen) at a concentration of 1 u/μl. The RNA concentration and integrity were assessed with an Agilent Technologies Bioanalyzer using a RNA 6000 Nano kit (Additional file 2: Note S2 and Figure S12). The RNase A-treated samples with an RIN value of 2.3 were used for miRNA expression analysis using TaqMan-based real-time PCR quantification (Applied Biosystems) (Additional file 2: Supplementary Methods) and small RNA sequencing (Illumina). One microgram of RNase A-treated total RNA was used for small RNA library preparation.

### In-house small RNA library preparation

Total RNA was isolated using TRIzol reagent (Ambion). RNA integrity was estimated with Agilent Technologies Bioanalyzer using RNA 6000 Nano kit (Agilent). One microgram of HEK-293T total RNA was used for standard small RNA library preparation using TruSeq small RNA kit v2 (Illumina) and prepared as described in the manufacturer’s protocol (Illumina). Fifty nanograms of HEK-293T total RNA was used for preparation of low input small RNA libraries. In brief, total RNA was ligated to the RNA 3′ and 5′ adapters (Illumina). The adapter-ligated small RNAs were reverse transcribed into cDNA using SuperScript II (Invitrogen) and PCR-amplified by 12 cycles using the PML PCR master mix (Illumina) with the TruSeq Small RNA 3′ indexed PCR primers (Illumina). After library amplification, samples were gel purified using a Novex TBE gel, 6% (Invitrogen) and 145–160-bp bands were excised, eluted, and ethanol precipitated. cDNA libraries were normalized to 4 nM, and single-end sequencing was carried out on an Illumina NextSeq 500.

Contaminated HEK-293T small RNA libraries were generated by pre-mixing HEK-293T total RNA with the total RNA isolated from *S2* cells. The following ratios of HEK-293T to *S2* total RNA were used 1000:1, 100:1, and 10:1.

### In-house library multiplexing and demultiplexing

The in-house prepared libraries (human HEK-293T *n* = 9 and mouse *n* = 9) were multiplexed and then sequenced in the same flow cell using an Illumina NextSeq500 high throughput sequencing v2 kit (75 cycles). The output data from Illumina sequencing machine were converted from BCL to FASTQ format and the pooled reads were de-multiplexed (assigned back to the original samples) based on the sample unique indices using bcl2fastq2 conversion software v 2.17. The demultiplexing process was performed three times: (1) using the default Illumina setting “--barcode-mismatches 1,” which allows one mismatch for index matching, (2) using a more stringent setting “--barcode-mismatches 0” to consider only perfect matches, (3) applying the same setting as in (2), but discarding the reads with inconsistent indices, which means that the index sequence reported by the main sequencing reaction (READ 1) is different from the index reported by the index sequencing reaction (INDEX 1) (Fig. 3e upper panel). The source to remove inconsistent indices can be found in the “scripts” folder of miRTrace package. The demultiplexed FASTQ files were pre-processed in the same way as the public datasets using miRTrace.

### Generating in silico mixture samples

To investigate the effect of cross-clade contamination on miRNA gene expression analysis and de novo miRNA prediction, in silico mixture samples with various levels of contamination were generated using four public data sets: pluripotent mouse embryonic stem cells (mESCs) cultivated in the media containing serum and leukemia inhibitory factor (LIF), the stimulated mESCs treated with retinoic acid (RA), the human monocyte-derived dendritic cells, and the fruit fly embryo (Additional file 3: Table S16). First, small RNA-Seq datasets of these samples were subsampled to the same sequencing depth of four million reads using seqtk (https://github.com/lh3/seqtk). Second, the mouse samples were contaminated with reads from the human and fruit fly samples to obtain various contamination levels: with present 0%, 0.0001%, 0.001%, 0.01%, 0.1%, 1%, 10%, 40%, 70%, and 100% spike-in reads. All of the mixture samples were processed for quality control using miRTrace. The output QC report is provided in Additional file 4: Reports S5–S6.

### Principal component analysis

The miRNA expression analysis was performed using miraligner from SeqBuster [45] with the command line “miraligner -sub 1 -trim 3 -add 1.” The *Mus musculus* miRNA precursor sequences (hairpin.fa) and relative mature miRNA coordinates (miRNA.str) from miRBase v21 were used as reference. The miRNA count table is available in Additional file 3: Tables S17–S18. The miRNA counts were normalized to read per million (RPM) based on the formula: (miRNA count/miRNA total counts per sample) × 10^6^. Principal component analysis was performed based on the sample covariance matrix of the miRNA expressions (RPM) using the prcomp() function without scaling in R v3.1.1. The PCA plots in 3D can be found in Additional file 2: Figures S7–S8.

### miRNA differential expression analysis

To identify the miRNAs that are differentially expressed (DE) in the stimulated mESCs (RA) with different levels of contaminations from 0 to 100% (RA 0–100%) versus the pluripotent mESCs without contaminations (LIF 0%), we used the following criteria. For miRNAs expressed in both samples, the miRNAs must be expressed at least ≥ 10 RPM in one of the samples and have > 2 fold change in RPM expression between the two samples. For miRNAs expressed only in one sample, the miRNA must be expressed at least ≥ 10 RPM. This is related to Additional file 2: Figures S9–S10.

### De novo miRNA prediction

Since the mouse miRNA precursors have been well mined, instead of identifying novel ones that are likely false positives, we measured how many known mouse miRNA precursors can be recovered by miRDeep2 analysis. The pluripotent mESC (LIF) samples with human and fruit fly spike-in reads were used for the analysis. The de novo miRNA prediction was performed independently for each sample in two steps using Perl scripts from miRDeep2 [28]. In the first step, the QC-passed reads (.fasta) were mapped to the mouse reference genome (mm10) using the command “mapper.pl -d -c -i -j -l 18 -m -p” to get the read mapping coordinates, which were deposited in an output file (with suffix .arf). In the second step, the novel precursor miRNAs were identified using the command “miRDeep2.pl -t Mouse.” The QC-passed reads (.fasta) and genome-wide mapping coordinates (.arf) from the first step were loaded as input, and the mouse reference genome (mm10), known rat mature miRNAs from miRBase v21, were used as related species reference.

Since the genome coordinates of novel miRNA precursors can vary depending on the read content of samples, the coordinates (with miRDeep2 score > 0) from each sample were pooled together and the overlapped coordinates were collapsed to get the maximum intervals (using bedtools merge v2.26.0), which were used to resolve coordinate inconsistency across samples. After annotating these precursors by intersecting with the coordinates of mouse precursors (mmu.gff3 from miRBase v21) using bedtool intersect v2.26.0, the numbers of known and unknown mouse precursors identified in each sample were counted.

### Sensitivity, specificity, and accuracy

For the miRNA differential expression analysis, the DE miRNAs that had been identified by comparing the miRNA profile of uncontaminated pluripotent to stimulated mESC samples (RA 0% vs. LIF 0%) were defined as true cases, and the non-DE miRNAs were defined as false cases. If the DE miRNAs (or true miRNAs), identified in the uncontaminated samples were still differentially expressed in the contaminated samples (RA 0.0001 - 70% vs. LIF 0%), these cases were considered to be true positives (TP); otherwise, they were considered to be false negatives (FN). Similarly, the previously identified non-DE miRNAs (or false miRNAs) that were still non-DE in the contaminated samples were considered to be true negatives (TN); otherwise, they were considered to be false positives (FP). For each sample, we calculated the sensitivity or true positive rate TPR = TP/(TP + FN), the specificity (SPC) or true negative rate SPC = TN/(TN + FP) and the accuracy ACC = (TP + TN)/(TP + FP + FN + TN). These values can be found in Additional file 3: Table S19.

For the de novo miRNA prediction, we defined the identified mouse miRNA precursors in the uncontaminated pluripotent mESC sample (LIF 0%) to be true cases, and the non-mouse precursors to be false cases. Based on whether the true or false precursors were identified in the contaminated samples (LIF 0.0001–70%), the TP, FN, TN, and FP were calculated in the same way as described above. The TPR, SPC, and ACC were also calculated using the same formulas. These values can be found in Additional file 3: Table S20.

### Tracing 12 *Drosophila* sequencing datasets to the species level

To demonstrate that miRTrace can be extended to trace sample species origins, we first obtained the reference catalog of species-specific miRNAs from Mohammed et al. 2018 [29] by parsing the “Supplemental_12flies_website.zip,” where the miRNA precursors with high confidence and that are only expressed in one species were considered as species-specific. In addition to the requirement of a species-specific expression pattern, it was also a condition that miRNA precursors should not have ortholog sequences in another species. The mature sequences of the qualified miRNA precursors were used as reference sequences. We then downloaded sRNA-Seq datasets from 12 *Drosophila* species from the NCBI SRA database (Additional file 3: Table S22). Since limited sRNA-Seq datasets are available from *Drosophila* species except for *D. melanogaster*, among the 12 samples only four of them are not used redundantly in the study by Mohammed et al. We mapped the reads of each sample to the collected species-specific miRNA sequences. A read is identified as species-specific miRNA if its first 20 nucleotides have an exact match to the first 20 nucleotides of the reference sequences.

## Additional files

Additional file 1: The catalog of clade-specific families. (TXT 3 kb)

Additional file 2: Supplementary notes, supplementary methods, and supplementary figures. (PDF 2180 kb)

Additional file 3: Supplementary tables. (XLSX 735 kb)

Additional file 4: Supplementary miRTrace reports. Report S1. miRTrace Trace report of the samples used in Fig. 2. Report S2. miRTrace QC report of the public *M. musculus* small RNA-Seq datasets used in Fig. 3a. Report S3. miRTrace QC report of the public *C. elegans* small RNA-Seq datasets used in Fig. 3a. Report S4. miRTrace QC report of the public *D. melanogaster* small RNA-Seq datasets used in Fig. 3a. Report S5. miRTrace QC report of the mouse samples in silico contaminated with various amounts of human sequences. The same samples as in main Fig. 3f left panel. Report S6. miRTrace QC report of the mouse samples in silico contaminated with various amounts of fruit fly sequences. The same samples as in main Fig. 3f right panel. Report S7–S8. miRTrace QC report of the samples used in Additional file 2: Figure S4. (ZIP 2285 kb)

Additional file 5: Review history. (DOCX 281 kb)

## References

[1] Arenas M, Pereira F, Oliveira M, Pinto N, Lopes AM, Gomes V, Carracedo A, Amorim A (2017). Forensic genetics and genomics: much more than just a human affair. PLoS Genet.

[2] Kircher M, Sawyer S, Meyer M (2012). Double indexing overcomes inaccuracies in multiplex sequencing on the Illumina platform. Nucleic Acids Res.

[3] Woese CR, Fox GE (1977). Phylogenetic structure of the prokaryotic domain: the primary kingdoms. Proc Natl Acad Sci U S A.

[4] Hebert PD, Cywinska A, Ball SL, deWaard JR (2003). Biological identifications through DNA barcodes. Proc Biol Sci.

[5] Galtier N, Nabholz B, Glemin S, Hurst GD (2009). Mitochondrial DNA as a marker of molecular diversity: a reappraisal. Mol Ecol.

[6] Yassin A, Markow TA, Narechania A, O'Grady PM, DeSalle R (2010). The genus Drosophila as a model for testing tree- and character-based methods of species identification using DNA barcoding. Mol Phylogenet Evol.

[7] Bartel DP (2009). MicroRNAs: target recognition and regulatory functions. Cell.

[8] Moran Y, Agron M, Praher D, Technau U (2017). The evolutionary origin of plant and animal microRNAs. Nat Ecol Evol.

[9] Sempere LF, Cole CN, McPeek MA, Peterson KJ (2006). The phylogenetic distribution of metazoan microRNAs: insights into evolutionary complexity and constraint. J Exp Zool B Mol Dev Evol.

[10] Deline B, Greenwood JM, Clark JW, Puttick MN, Peterson KJ, Donoghue PCJ (2018). Evolution of metazoan morphological disparity. Proc Natl Acad Sci U S A.

[11] Tarver JE, Sperling EA, Nailor A, Heimberg AM, Robinson JM, King BL, Pisani D, Donoghue PC, Peterson KJ (2013). miRNAs: small genes with big potential in metazoan phylogenetics. Mol Biol Evol.

[12] Kenny NJ, Sin YW, Hayward A, Paps J, Chu KH, Hui JH (2015). The phylogenetic utility and functional constraint of microRNA flanking sequences. Proc Biol Sci.

[13] Fromm B, Billipp T, Peck LE, Johansen M, Tarver JE, King BL, Newcomb JM, Sempere LF, Flatmark K, Hovig E, Peterson KJ (2015). A uniform system for the annotation of vertebrate microRNA genes and the evolution of the human microRNAome. Annu Rev Genet.

[14] Tarver JE, Taylor RS, Puttick MN, Lloyd GT, Pett W, Fromm B, Schirrmeister BE, Pisani D, Peterson KJ, Donoghue PCJ (2018). Well-annotated microRNAomes do not evidence pervasive miRNA loss. Genome Biol Evol.

[15] Thomson RC, Plachetzki DC, Mahler DL, Moore BR (2014). A critical appraisal of the use of microRNA data in phylogenetics. Proc Natl Acad Sci USA.

[16] Berezikov E (2011). Evolution of microRNA diversity and regulation in animals. Nat Rev Genet.

[17] Peterson KJ, Dietrich MR, McPeek MA (2009). MicroRNAs and metazoan macroevolution: insights into canalization, complexity, and the Cambrian explosion. Bioessays.

[18] Taylor RS, Tarver JE, Hiscock SJ, Donoghue PC (2014). Evolutionary history of plant microRNAs. Trends Plant Sci.

[19] Hennig W (1950). Grundzuge einer Theorie der phylogenetischen Systematik.

[20] Kozomara A, Griffiths-Jones S (2014). miRBase: annotating high confidence microRNAs using deep sequencing data. Nucleic Acids Res.

[21] Faridani OR, Abdullayev I, Hagemann-Jensen M, Schell JP, Lanner F, Sandberg R (2016). Single-cell sequencing of the small-RNA transcriptome. Nat Biotechnol.

[22] Buck AH, Coakley G, Simbari F, McSorley HJ, Quintana JF, Le Bihan T, Kumar S, Abreu-Goodger C, Lear M, Harcus Y (2014). Exosomes secreted by nematode parasites transfer small RNAs to mammalian cells and modulate innate immunity. Nat Commun.

[23] Wright ES, Vetsigian KH (2016). Quality filtering of Illumina index reads mitigates sample cross-talk. BMC Genomics.

[24] Matranga CB, Andersen KG, Winnicki S, Busby M, Gladden AD, Tewhey R, Stremlau M, Berlin A, Gire SK, England E (2014). Enhanced methods for unbiased deep sequencing of Lassa and Ebola RNA viruses from clinical and biological samples. Genome Biol.

[25] Licatalosi DD, Mele A, Fak JJ, Ule J, Kayikci M, Chi SW, Clark TA, Schweitzer AC, Blume JE, Wang X (2008). HITS-CLIP yields genome-wide insights into brain alternative RNA processing. Nature.

[26] Hafner Markus, Landthaler Markus, Burger Lukas, Khorshid Mohsen, Hausser Jean, Berninger Philipp, Rothballer Andrea, Ascano Manuel, Jungkamp Anna-Carina, Munschauer Mathias, Ulrich Alexander, Wardle Greg S., Dewell Scott, Zavolan Mihaela, Tuschl Thomas (2010). Transcriptome-wide Identification of RNA-Binding Protein and MicroRNA Target Sites by PAR-CLIP. Cell.

[27] Terranova C, Narla ST, Lee YW, Bard J, Parikh A, Stachowiak EK, Tzanakakis ES, Buck MJ, Birkaya B, Stachowiak MK (2015). Global developmental gene programing involves a nuclear form of fibroblast growth factor receptor-1 (FGFR1). PLoS One.

[28] Friedlander MR, Mackowiak SD, Li N, Chen W, Rajewsky N (2012). miRDeep2 accurately identifies known and hundreds of novel microRNA genes in seven animal clades. Nucleic Acids Res.

[29] Mohammed J, Flynt AS, Panzarino AM, Mondal MMH, DeCruz M, Siepel A, Lai EC (2018). Deep experimental profiling of microRNA diversity, deployment, and evolution across the Drosophila genus. Genome Res.

[30] Wingett SW, Andrews S (2018). FastQ screen: a tool for multi-genome mapping and quality control. F1000Res.

[31] Lagos-Quintana M, Rauhut R, Lendeckel W, Tuschl T (2001). Identification of novel genes coding for small expressed RNAs. Science.

[32] Lau NC, Lim LP, Weinstein EG, Bartel DP (2001). An abundant class of tiny RNAs with probable regulatory roles in Caenorhabditis elegans. Science.

[33] Lee RC, Ambros V (2001). An extensive class of small RNAs in Caenorhabditis elegans. Science.

[34] Philip A, Ferro VA, Tate RJ (2015). Determination of the potential bioavailability of plant microRNAs using a simulated human digestion process. Mol Nutr Food Res.

[35] Pirritano M, Fehlmann T, Laufer T, Ludwig N, Gasparoni G, Li Y, Meese E, Keller A, Simon M (2018). Next generation sequencing analysis of total small noncoding RNAs from low input RNA from dried blood sampling. Anal Chem.

[36] Keller A, Kreis S, Leidinger P, Maixner F, Ludwig N, Backes C, Galata V, Guerriero G, Fehlmann T, Franke A (2017). miRNAs in ancient tissue specimens of the Tyrolean iceman. Mol Biol Evol.

[37] Reinhart BJ, Bartel DP (2002). Small RNAs correspond to centromere heterochromatic repeats. Science.

[38] Argaman L, Hershberg R, Vogel J, Bejerano G, Wagner EG, Margalit H, Altuvia S (2001). Novel small RNA-encoding genes in the intergenic regions of Escherichia coli. Curr Biol.

[39] Pfeffer S, Zavolan M, Grasser FA, Chien M, Russo JJ, Ju J, John B, Enright AJ, Marks D, Sander C, Tuschl T (2004). Identification of virus-encoded microRNAs. Science.

[40] Ratnasingham S, Hebert PD (2007). bold: the barcode of life data system (http://www.barcodinglife.org). Mol Ecol Notes.

[41] Griffiths-Jones S, Saini HK, van Dongen S, Enright AJ (2008). miRBase: tools for microRNA genomics. Nucleic Acids Res.

[42] Juhling F, Morl M, Hartmann RK, Sprinzl M, Stadler PF, Putz J (2009). tRNAdb 2009: compilation of tRNA sequences and tRNA genes. Nucleic Acids Res.

[43] Quast C, Pruesse E, Yilmaz P, Gerken J, Schweer T, Yarza P, Peplies J, Glockner FO (2013). The SILVA ribosomal RNA gene database project: improved data processing and web-based tools. Nucleic Acids Res.

[44] Kang W, Bang-Berthelsen CH, Holm A, Houben AJ, Muller AH, Thymann T, Pociot F, Estivill X, Friedlander MR (2017). Survey of 800+ data sets from human tissue and body fluid reveals xenomiRs are likely artifacts. RNA.

[45] Pantano L, Estivill X, Marti E (2010). SeqBuster, a bioinformatic tool for the processing and analysis of small RNAs datasets, reveals ubiquitous miRNA modifications in human embryonic cells. Nucleic Acids Res.

[46] Kang W, Eldfjell Y, Fromm B, Estivill X, Biryukova I, Friedländer MR: miRTrace quality control of small RNA-Seq data prepared from low-input, degraded and contamianted HEK-293T RNA samples. Gene Expression Omnibus. GSE118437. https://www.ncbi.nlm.nih.gov/geo/query/acc.cgi?acc=GSE118437. Accessed 02 Nov 2018.

[47] Friedländer MR, Eldfjell Y, Kang W: miRTrace v1.0.0. Zenodo. 2018. https://zenodo.org/record/1479406. 10.1186/s13059-018-1588-9 PMC6280396 30514392

