# Supplementary material for: miRTrace reveals the organismal origins of microRNA sequencing data
Source: Genome Biol. 2018 Dec 4;19:213. doi: 10.1186/s13059-018-1588-9 (PMC6280396; doi:10.1186/s13059-018-1588-9)
Supplement: Supplementary file 2 — Supplementary notes, supplementary methods, and supplementary figures. (PDF 2180 kb) [file 13059_2018_1588_MOESM2_ESM.pdf]

SUPPLEMENTARY INFORMATION FOR

miRTrace reveals organismal origins of microRNA sequencing data

Wenjing Kang[1], Yrin Eldfjell[1], Bastian Fromm[1], Xavier Estivill[2], Inna Biryukova[1], Marc R. Friedländer[1]

[1] Science for Life Laboratory, Department of Molecular Biosciences, The Wenner-Gren Institute, Stockholm University, Stockholm, Sweden
[2] Genes and Disease Research Group, Sidra Medicine Research Center, Doha, Qatar

Table of Contents

Supplementary Notes 3
Note S1: Effects of miRNA annotation imbalances between species 3
Note S2: RNA integrity and small RNA sequencing quality 4

Supplementary Methods 5
Estimating sensitivity of contamination detection 5
Estimating specificity of clade-specific miRNA detection 5
RNA degradation at room temperature 6
TaqMan small RNA assays 6
Index mis-assignment analysis using in-house libraries 6
Computational removal of mis-assigned reads based on Matranga et al. 2014 7
Tracing species origins of simulated Sanger COI sequences using Barcode of Life Identification Engine 7
Tracing species origins of RNA-Seq datasets using FastQ Screen 8

Supplementary Figures 9
Figure S1: Consistency of clade-specific miRNAs reported using miRBase and MirGeneDB as reference database 10
Figure S2: Specificity of clade-specific miRNA detection 11
Figure S3: Presences of clade-specific miRNA families in public data sets 13
Figure S4: miRTrace sensitivity in detecting contamination from different clades 14
Figure S5: Circos plot showing potential index mis-assignments when allowing one mismatch in the index sequence 15
Figure S6: Comparison of two approaches for removing mis-assigned reads 16
Figure S7: PCA of mouse samples with human contamination 17
Figure S8: PCA of mouse samples with fruit fly contamination 18
Figure S9: miRNA differential expression analysis of mouse samples with human contamination 19
Figure S10: miRNA differential expression analysis of mouse samples with fruit fly contamination 20
Figure S11: Neighbor-joining tree of COI sequences of the 12 *Drosophila* species 21
Figure S12: RNA integrity assessment by RIN 22

References 23

Supplementary Notes

Note S1: Effects of miRNA annotation imbalances between species

In some of our analyses we have observed that the relative proportions of clade-specific miRNAs reported by miRTrace do not correspond to the known proportions of transcripts in the samples. For instance, in main Figure 5F, the reported fruit fly contaminations are 1-2 orders of magnitude larger than would be expected from known proportions of the mixed samples, suggesting that miRTrace detects miRNAs from distinct clades with different sensitivity.

To investigate the sensitivity with which miRTrace detects contaminations from distinct clades, a mouse data set was *in silico* contaminated with varying amount of sequences from 12 representative species, one species at the time (Additional file 4: Report S7). In general, miRTrace sensitively detects the contaminations from any of the species when present at 0.1% or higher, except for *Picea abies* from the gymnosperm clade (Figure S4). However, the exact detection sensitivity varies from clade to clade. For example, the contaminations from *Drosophila melanogaster* (insect clade), *Gallus gallus* (bird), *Amphimedon queenslandica* (dicot plant) and *Physcomitrella patens* (bryophyte plant) are detected when only 0.001% contaminations are present, while the presence of *Danio rerio* (fish) and *Apostichopus japonicas* (echinoderm) is not detected until 0.1% contaminations are present, and the same number for *Picea abies* (gymnosperm) is 10% contaminations.

One possible reason for this is that some clades do not have many annotated clade-specific miRNAs. For instance, there are currently only six annotated miRNA families that are specific to gymnosperms, while sixty miRNA families are known to be specific to insects. These imbalances in part reflect the resources put into annotation efforts, for instance well-studied model organisms like fruit flies versus gymnosperm plants. Therefore, an effort to make a uniform miRNA annotation across animals and plants will likely ensure more quantitative analyses by miRTrace.

Another possible reason for the varying sensitivity of our method is that some clade-specific miRNAs may be generally highly expressed, while other might be generally lowly expressed. This can be seen by contrasting miRNAs that are specific to gymnosperm plants to those that are specific to nematodes (Additional file 4: Report S8). As mentioned, there are six miRNA families that are specific to gymnosperms; these are present at 7 reads per million (RPM) in the gymnosperm data used in Figure S4. Comparably, there are just four families that are specific to nematodes, however these are present at >18,000 RPM in the nematode data. Since it is well established that miRNA families can differ largely in their overall levels of expression, these differences likely reflect biology.

In summary, the varying sensitivity stems in part from the fact that miRTrace integrates information from a variable number of miRNA gene loci, rather than relying on information from a single barcoding gene locus. However, as discussed in the main manuscript, this makes the analyses more robust to evolutionary events such as gene losses. Similarly, miRTrace profiles RNA molecules, which can differ in copy number per cell, rather than DNA molecules that are present at constant levels per cell (typically two copies for diploid organisms). Since RNA molecules are often present in individual cells in copy numbers of hundreds or thousands, this makes the method sensitive, so that it can accurately assign single cells to their clade of origin (see main Figure 2B).

Note S2: RNA integrity and small RNA sequencing quality

Quality and quantity of starting RNA material are the essential factors determining the accuracy of sequencing outcome. Ribosomal RNA comprises > 80 % of total RNA sample and can be easily and accurately quantified compared to mRNA and small RNAs, which make up < 7 % of total RNAs. Traditionally, the RNA integrity number (RIN) measured by evaluating ribosomal RNA quality is broadly used to indicate the quality of other RNA species. RNA samples with an RIN value ≥ 8 are recommended for small RNA library preparation (Illumina, Diagenode and Qiagen small RNA NGS protocols). In practice, RIN values ranging from 10.00 to 7.00 are usually considered as good quality starting material by NGS facilities.

In our hands, small RNA libraries prepared from total RNAs degraded by RNase A enzyme with a low RIN value (2.30, main Figure 5: 7th bar) showed the miRNA outcome comparable with the standard small RNA libraries (high RIN value 10.00-9.00, main Figure 5: the 5th bar). This result supports the previously finding of robust miRNA stability in degraded clinical samples, which was measured by RT-qPCR[1]. Importantly, there is no correlation between RIN value and degradation of small RNAs. This is especially relevant to clinical and field-collected samples, which are particularly subject to RNA degradation. We suggest that the samples with minimal RIN value of 2.00-2.50 could be used for small RNA library preparation using standard TruSeq small RNA protocol (Illumina).

There are a number of controversial reports on miRNA stability at different storage conditions [1-3]. We re-evaluated RNA stability at room temperature over period of 10 days in air-protected and air-exposed RNA samples isolated from HEK-293T with a standard TRIzol-based protocol (Figure S12). In our hands, air-protected RNA samples were stable at room temperature without significant degradation (RIN 10.00-9.60). In contrast, air-exposed RNA samples showed a slight sign of RNA degradation (with RIN values decreasing from 10.00 to 8.20). This suggests that short-term storage of total RNA samples at room

temperature does not necessarily cause a substantial decrease of RNA quality, as measured by rRNA integrity.

Supplementary Methods

Estimating sensitivity of contamination detection

To investigate the sensitivity of miRTrace, in detecting contaminations from different species, a mouse sample (SRX869508) was computationally contaminated with reads from 11 species. These various levels of contaminations were tested: 0.0001%, 0.001%, 0.01%, 1% and 10% (Additional file 4: Report S7). The samples that serve the contaminating "spike-in" reads are from the following species: *Physcomitrella patens* (SRR768442), *Picea abies* (SRR1771544), *Oryza sativa* (SRR1013788), *Arabidopsis thaliana* (SRX1629219), *Amphimedon queenslandica* (SRR014252), *Caenorhabditis elegans* (SRX748227), *Drosophila melanogaster* (SRX718006), *Crassostrea gigas* (SRR317146), *Apostichopus japonicas* (SRR934651), *Danio rerio* (SRX529157), *Gallus gallus* (ERX1266892) and *Homo sapiens* (SRX808067). Beside this, to study the sensitivity of detecting mouse contaminations, we also spiked in reads from the mouse sample to the *Arabidopsis thaliana* sample. These samples were subsampled to the same sequencing depth of four million reads using seqtk v 1.2 (http://github.com/lh3/seqtk). These samples were then processed to profile clade-specific miRNAs using miRTrace. The method part is related to Figure S4.

Estimating specificity of clade-specific miRNA detection

To estimate how many reads are likely to be identified as clade-specific miRNAs by chance, a binomial model was adopted for the analysis. The Bernoulli trails $\boldsymbol{X} = (X_1, X_2 \dots X_n)$ of detecting clade-specific miRNA were assumed to be independent and identical distributed (iid) and follow binomial distribution. The probability of detecting exactly $k\ (k = 0,1,2,3 \dots n)$ out of n clade-specific miRNAs was given by the probability mass function:

$$f(k; n, p) = \Pr(X = k) = \binom{n}{k} p^k (1-p)^{n-k},$$

Where $p = \left(\frac{1}{4}\right)^{20}$ was the probability of matching two sequences (coded in ATCG; 20 nts) by chance. According to the probability mass function, probability of each read being identified as clade-specific miRNAs by chance was $\Pr(\mathrm{X} > 0) = 1 - f(0; n; p)$. Suppose the experiments $\boldsymbol{Y} = (Y_1, Y_2 \dots Y_r)$ of matching reads to the reference sequences were also binomially distributed. Given r number of sequencing reads in a library and n number of unique clade-specific miRNAs as reference, on average $\mathrm{E}[\boldsymbol{Y}] = r \times \Pr(\mathrm{X} > 0)$ reads are

expected to be detected as clade-specific miRNA by chance. The method part is related to Figure S2.

RNA degradation at room temperature

10 µg of total RNA samples were incubated at room temperature for up to 10 days either in the lid-closed tubes (air-protected) or in the tubes without lids (air-exposed). At each time point, the tubes were removed from room temperature and stored at -80°C until analysis. As control, total RNA samples stored at -80°C were used. Aliquots corresponding to 50-100ng of the initial RNA amounts were heat denatured for 2 min at 70°C and analyzed on a Bioanalyzer using RNA 6000 Nano kit. The degradation kinetics of ribosomal RNA was assessed by the RIN. The method part is related to Figure S12.

TaqMan small RNA assays

10 ng of RNA from each samples was reverse transcribed into cDNA using a custom TaqMan miRNA RT kit. RNAse/DNAse free water and the RT reaction without reverse transcriptase were used as negative controls. cDNA from the samples and negative controls were analyzed using custom TaqMan small RNA assays ID 00037 and ID 002299 targeting hsa-miR-10a and hsa-miR-191, respectively. TaqMan assays were performed on an OneStep Plus thermocycler, according to the manufacturer's protocol. Cycling conditions: activation 10 min 95°C, cycling 15 s 95°C, 60 s 60°C for 40 cycles. The temperature-dependent increases of Ct values was observed between samples after 1 min of incubation with RNase A. The Ct values of the samples incubated at +4°C and +30°C were 28.38±0.025 and 29.72±0.19, respectively for hsa-miR-10a and 28.72±0.09 and 29.99+0.19 for hsa-miR-191.

Index mis-assignment analysis using in-house libraries

During demultiplexing, reads can be mis-assigned to the wrong sample if errors occur in the index sequence. This happened in our in-house libraries and was detected as cross-clade contaminations by miRTrace since rodent-specific miRNAs were mis-assigned to the human samples (main Figure 3C). To check if the index mis-assignment is more likely to happen in the samples with similar indices, we generated a network to show the similarity of indices across samples (Figure S5). We found the samples with more similar indices (or links) tend to have more mis-assigned reads, which is consistent with main Figure 3C.

We performed this analysis using the in-house samples that were demultiplexed with allowing 1 mismatch for index matching. We first extracted index sequence of each read using Linux "grep" command. Each FASTQ read is encoded by 4 lines: first is ID line, second is raw sequence, third is a symbol "+" and fourth has quality values for the sequence. In our case, the index sequence is located in the second field of the ID line, which is separated by

space. For each sample, we then collapsed the redundant indices to unique sequences. As expected, each sample has 25 index possibilities: the default index and plus 24 indices with one mismatch to the default one. Each index was then aligned to any other index from other samples. If they differ by 1 nucleotide using pairwiseAlignment() function from Biostrings package in R v 3.0.1, we defined them as an index pair. The network of potential mis-assigned cases was generated based on these identified index pairs. The method part is related to Figure S5.

Computational removal of mis-assigned reads based on Matranga et al. 2014

To compare how well our approach (in main Figure 3E) removing mis-assigned reads compare to the method used in Matranga et al. 2014 [4], we processed our raw sequencing data based on the cutoffs suggested by the method section "Demultiplexing of sequencing runs and QC" of Matranga's paper [4]. In brief, the output data from Illumina sequencing machine were demultiplexed from BCL to FASTQ format based on the sample unique indices using bcl2fastq v 2.17 with option "--barcode-mismatches 0 --create-fastq-for-index-reads". In addition to the demultiplexed FASTQ file for reads, a separate FASTQ file with the corresponding index information was generated, containing index identity (same as the read identity), index sequence and index quality scores. To remove the reads with low-quality indices, we first discarded the low-quality indices that have a minimum quality score less than Q25 using fastq_quality_filter with option "-q 25 -p 100" from Fastx v 0.0.14. The identities of the remained indices were then used to extract the qualified reads using seqtk subseq. The method part is related to Figure S6.

Tracing species origins of simulated Sanger COI sequences using Barcode of Life Identification Engine

The aim of the analysis is to know how well miRTrace tracing taxonomic origins of sample compare to Barcode of Life Identification Engine [5], which uses certain genes in an organism as marker to identify species origin. For example the mitochondrial gene cytochrome oxidase I (COI) gene is used as barcode for animal species. Sanger sequencing is widely used in the scientific community to profile the DNA barcode region for species analysis. In order to mimic the real data, we simulated COI sequences of the 12 *Drosophila* species (same as in main Figure 6) with taking into account intra-species variation and Sanger sequencing error and then applied Barcode of Life Identification Engine to resolve species origins of these sequences.

To obtain COI sequences of the 12 *Drosophila* species as reference for simulation, we aligned *Drosophila melanogaster* COI sequence (with GeneBank accession number: HM102299) to nucleotide collection (nr/nt) database with specifying the organism option as the other 11 *Drosophila* species using nucleotide BLAST

(https://blast.ncbi.nlm.nih.gov/Blast.cgi). The COI sequences of the other 11 *Drosophila* species were manually selected based on the best blast alignments. The intra-species mutation rate was calculated based on the pairwise alignments of *Drosophila persimilis* COI sequences downloaded from barcode of life data system (BOLD system http://www.boldsystems.org/). For each pairwise alignment, we measured how different between the sequences by dividing the number of mismatches by the alignment length. Among these measurements, the maximum value 0.013 (13 mismatches in 1000 nucleotides) was used as intra-species mutation rate for all the 12 species. For each species, we simulated 1000 sequences from the corresponding COI sequence with considering both intra-species variation and Sanger error. We used custom script to introduce intra-species variation, while Mason v 0.1.2 [6] was applied to introduce Sanger sequencing error. To resolve species origin, each simulated sequence was mapped against to the 7,312 *Drosophila* COI records (downloaded from BOLD system, 20/08/2018), which have species names and represent more than 400 *Drosophila* species, using blastn in BLAST v 2.2.31. We defined the good blast alignments primarily by high bit-score and secondarily by low e-value. If sequence matches equally well to more than one species, we marked it as "ambiguous hit". The method part is related to main Figure 6B.

Tracing species origins of RNA-Seq datasets using FastQ Screen

FastQ Screen [7] is originally built for quality control of RNA-Seq datasets, especially for contamination detection, but can also be used to characterize species origins of sample. It aligns sequencing reads against a panel of different genomes provided by user to resolve from where the sequences originate. In order to make a fair comparison between miRTrace and FastQ Screen, we used public RNA-Seq datasets from the 12 *Drosophila* species in main Figure 6. Notably, three of them (*D.melanogaster*: SRR4463849, *D.erecta*: SRR1617567 and *D.virilis*: SRR1617568) are from the same studies as the miRNA sequencing data shown in main Figure 6A. The SRA number of these datasets can be found in Additional file 3: Table S23. We used 12 *Drosophila* genomes downloaded from FlyBase, including *D.simulans* (r2.02), *D.sechellia* (r1.3), *D.melanogaster* (r6.22), *D.yakuba* (r1.05), *D.erecta* (r1.05), *D.ananassae* (r1.05), *D.pseudoobscura* (r3.04), *D.persimilis* (r1.3), *D.willistoni* (r1.05), *D.mojavensis* (r1.04), *D.virilis* (r1.06) and *D.grimshawi* (r1.05), as reference. The RNA-Seq datasets were mapped against to each of the 12 reference genomes using FastQ Screen v 0.9.2 with option "--aligner bowtie --subset 100000". The "one hit/one genome" and "multiple hits/one genome" reads were used for the species analysis. The method part is related to main Figure 6C.

Supplementary Figures

A) Comparison of clade-specific miRNA counts profiled using miRBase and MirGeneDB

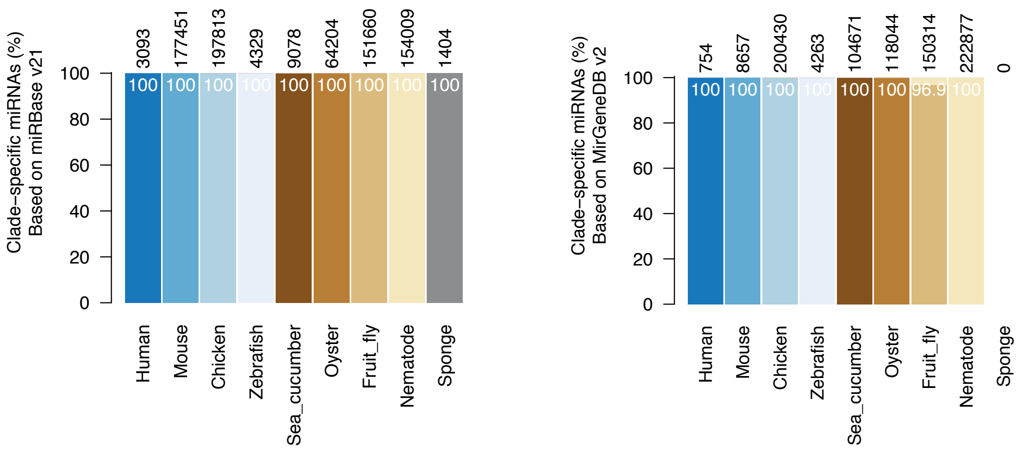

B) Comparison of clade-specific miRNA families profiled using miRBase and MirGeneDB

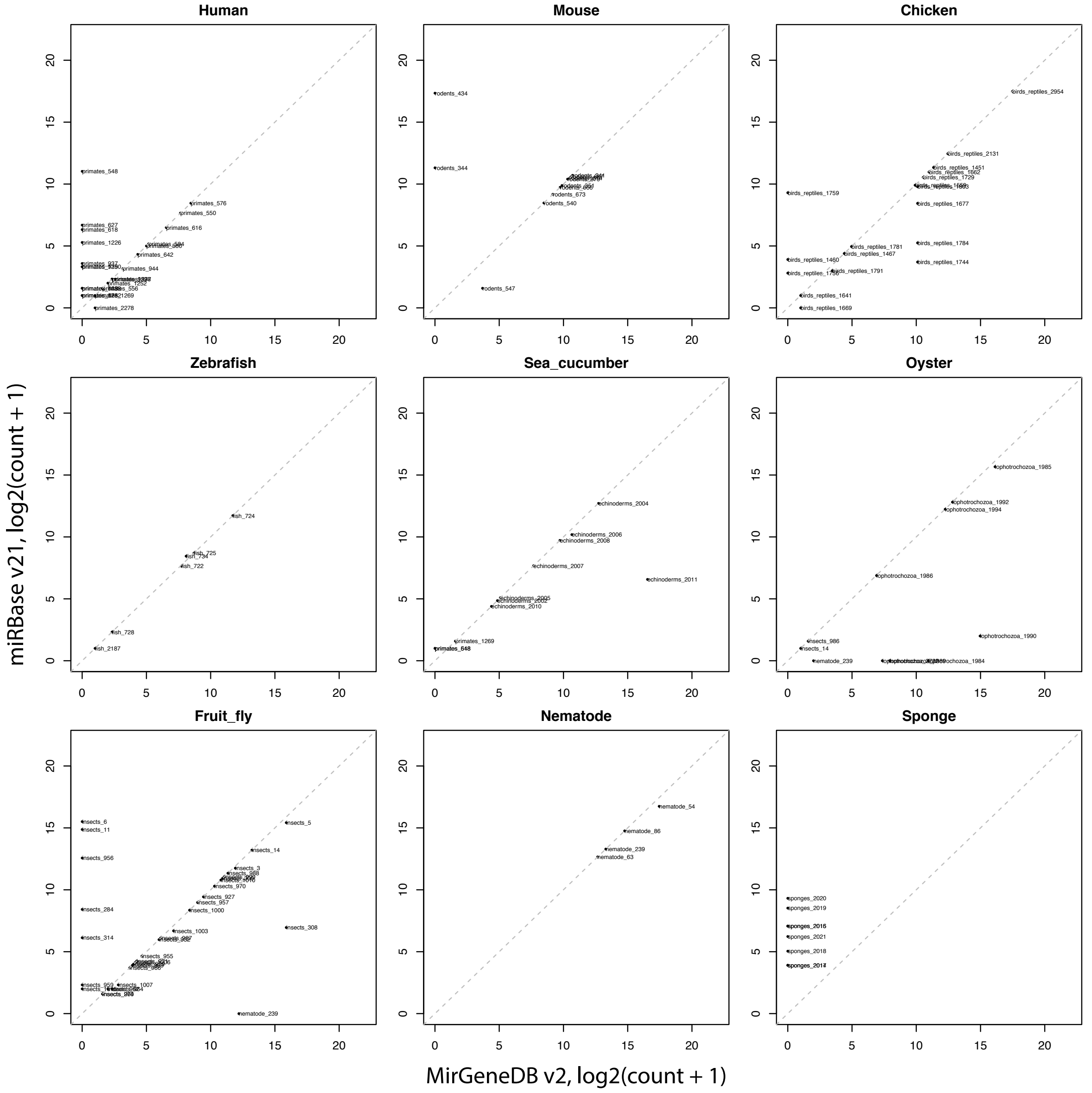

Figure S1: Consistency of clade-specific miRNAs reported using miRBase and MirGeneDB as reference database

A) The bar plot shows the composition of clade-specific miRNAs profiled using miRBase and MirGeneDB. These are the same samples as used in main Figure 2A. The number on the top of each bar indicates the total number of clade-specific miRNAs. The white number inside the bar indicates the proportion of the clade-specific miRNAs that are assigned to the expected clade. Notably, MirGeneDB does not have miRNA entries for sponge (right panel, the 9th bar).

B) The scatter plot shows the consistency of the abundance of clade-specific miRNA family profiled using miRBase and MirGeneDB. For each sample, the family abundance is calculated by summing up the counts of the miRNAs belonging to the family. The families on the diagonal line are equally abundant, indicating miRBase and MirGeneDB give consistent miRNA sequences as reference. The families that are off the diagonal into the upper and lower triangular region tend to have more miRNA sequences as reference from miRBase and MirGeneDB respectively. For example, the miRNA families on the vertical x=0 have miRNA entries in miRBase but not in MirGeneDB, indicating these sequences could be newly emerged miRNAs or false positives.

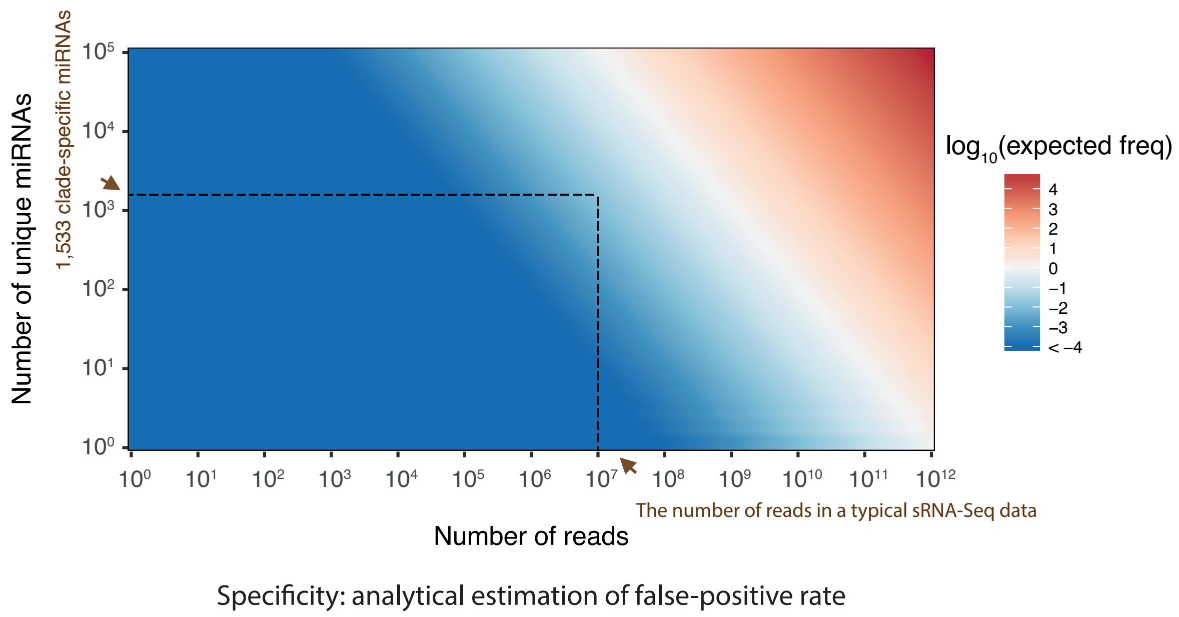

Figure S2: Specificity of clade-specific miRNA detection

The plot shows the absolute number of clade-specific miRNAs detected by chance as a function of sequencing depth (number of reads) and number of unique miRNAs in the reference database. The estimations are based on binomial statistics, as described above in the Supplementary Method section "Estimating specificity of clade-specific miRNA detection". The blue, white and red colors indicate the expected absolute frequency is less than 1, equal to 1 and more than 1 respectively. No miRNA sequence (with expected absolute frequency < 1) is expected to be detected by chance given a typical sRNA-Seq sequencing depth with 10 million reads and 1,533 unique clade-specific miRNAs used in the study (arrowheads and dashed lines). More information of the analysis can be found in Supplementary Method section "Estimating specificity of clade-specific miRNA detection".

Public sRNA-Seq data sets

Mouse (n = 428)

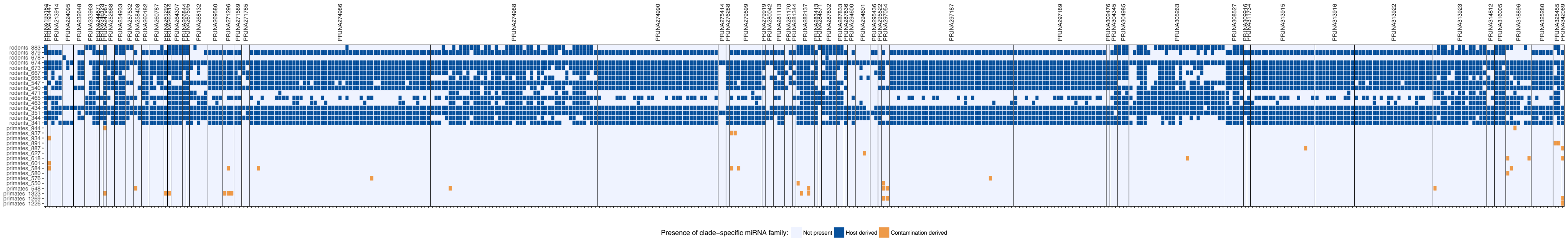

Nematode (n = 151)

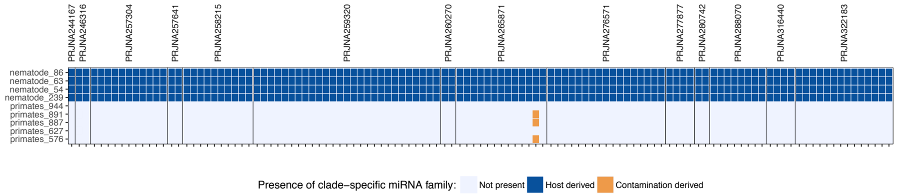

Drosophila (n = 150)

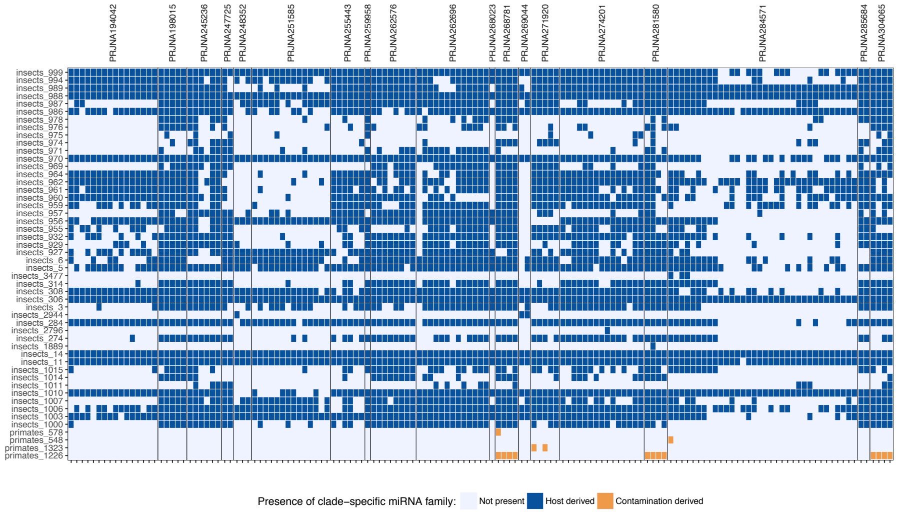

Figure S3: Presences of clade-specific miRNA families in public data sets

The heatmaps show the presences of the clade-specific miRNA families. Every column represents one sample. The samples are grouped in studies based on NCBI Bio project numbers, which are showed on the top of the heatmaps. Every row indicates a clade-specific miRNA family. The host derived clade-specific miRNAs are widely present across studies, while most of the contamination-derived clade-specific miRNAs are sparsely distributed, and some of them are present in study-specific manner, indicating batch effect. The miRTrace reports of these public data sets can be found in Additional file 4: Report S2 - S4.

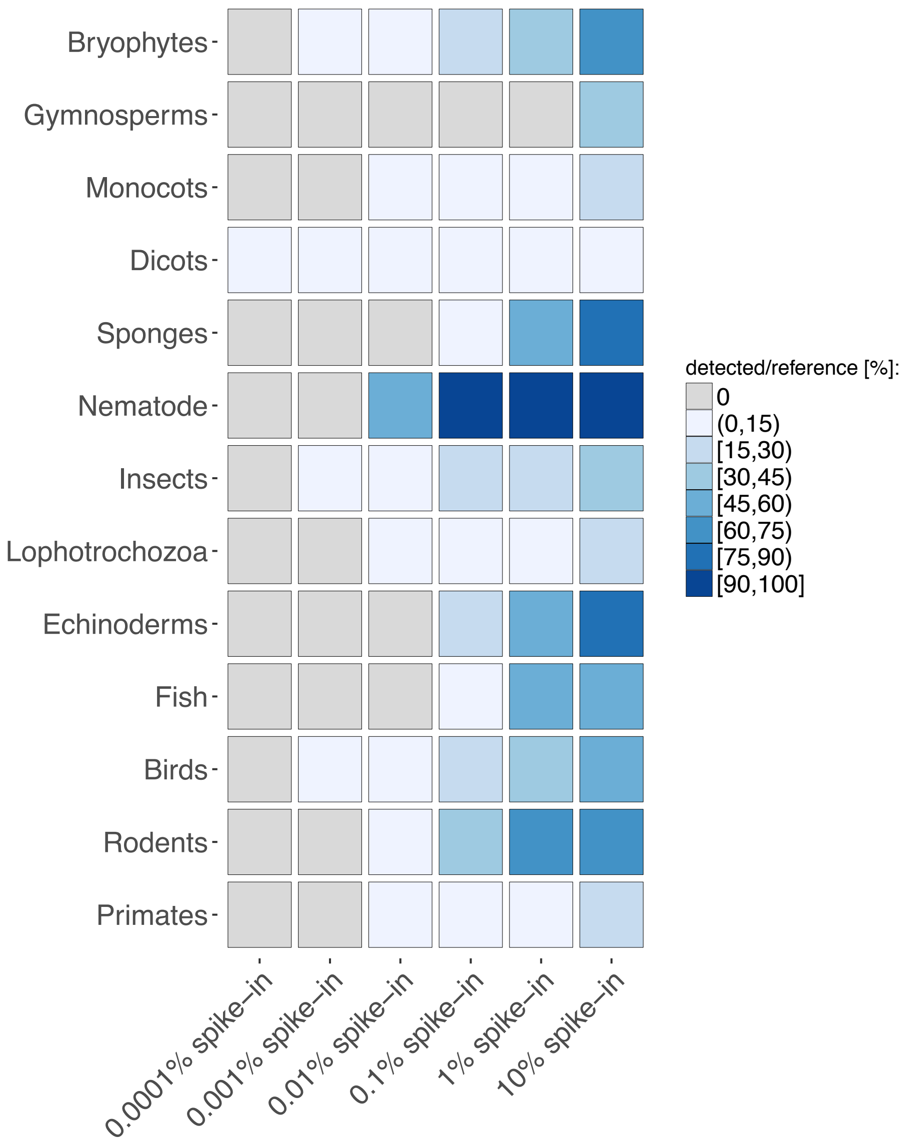

Figure S4: miRTrace sensitivity in detecting contamination from different clades

The heatmap shows how many proportion of reference clade-specific miRNA families are detected in the samples with various amounts of contamination reads from 13 clades. To see the exact species used for the analysis, see the Supplementary Method section "Estimating sensitivity of contamination detection".

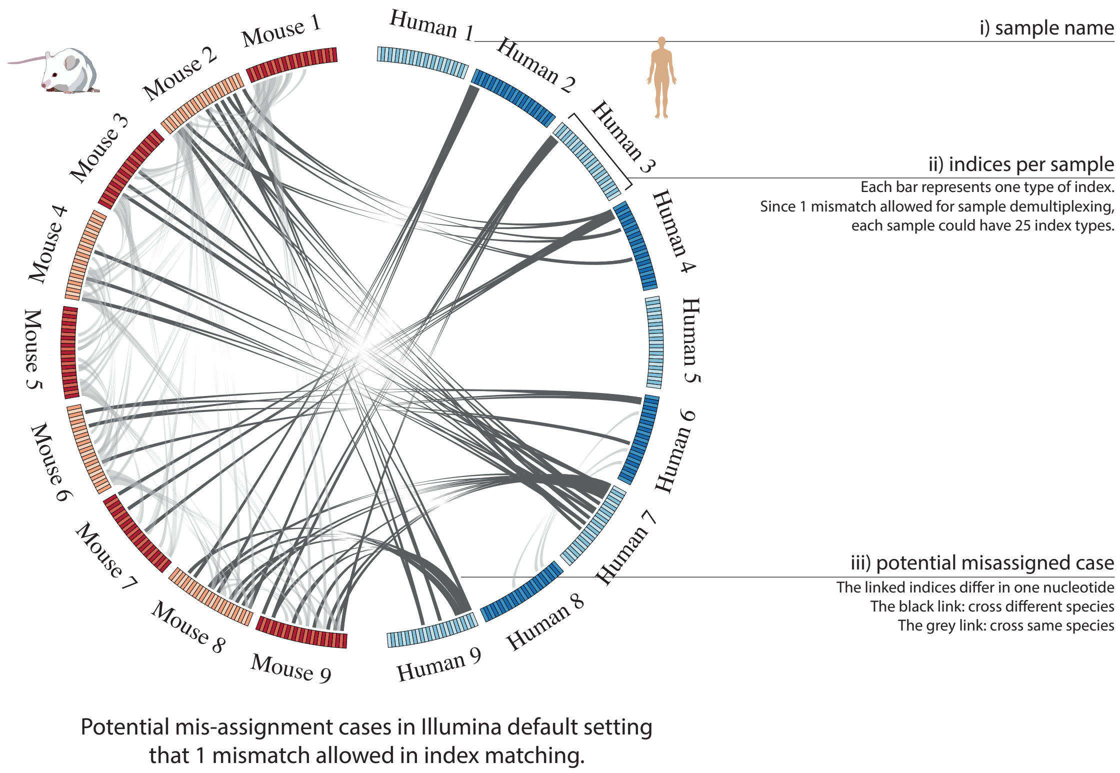

Figure S5: Circos plot showing potential index mis-assignments when allowing one mismatch in the index sequence

The outer ring shows the nine human (blue) and nine mouse samples (red) that were used for the experiment in main figure 3C-E. The next ring represents the possible indices of each sample, allowing for one mismatch. Each sample can thus have 25 different indices: the expected (correct) index and 24 that results from sequencing errors at various positions. There are 24 possible indices assuming a single mismatch error, since the index is 6 nucleotides long, and a sequencing error can occur by any of these positions being converted into one of the three wrong nucleotides or an 'N', representing an uncalled nucleotide. The links show indices that are differ in one nucleotide between different samples. These are cases where a single sequencing error can cause an index to be assigned to the wrong sample. Interestingly, the human samples 7 and 9 have the most links. These are samples that have high contamination rates, but where the contamination disappears when only perfect index matches are considered (main Figure 3C-D). More information about the analysis can be found in Supplementary Method section "Index mis-assignment analysis using in-house libraries

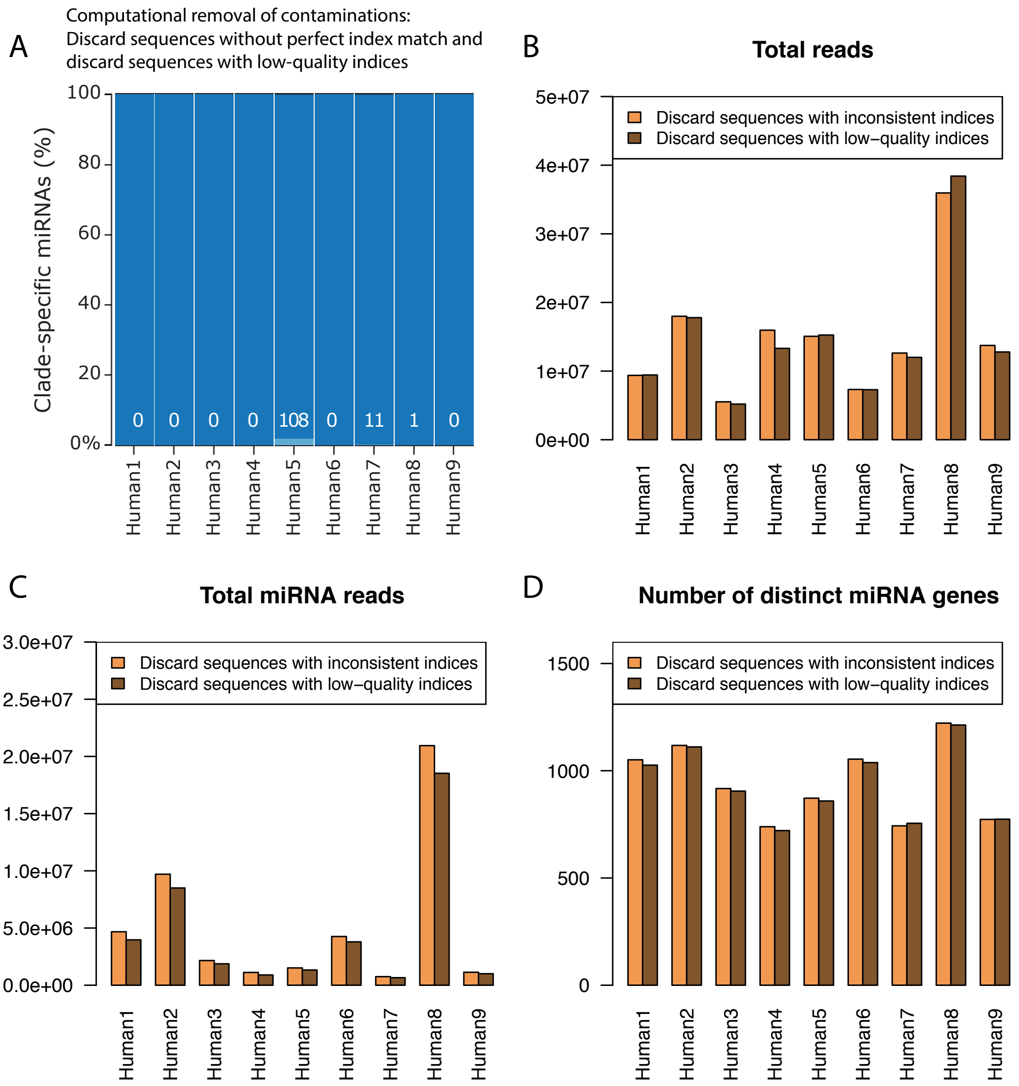

Figure S6: Comparison of two approaches for removing mis-assigned reads

Human and mouse samples were sequenced together in the same flow cell. Two approaches were used for sample demultiplexing and QC check: 1) Demultiplexing with allowing for 0 mismatches and discarding sequences with inconsistent indices (see main Figure 3E); 2) Demultiplexing with allowing for 0 mismatches and discarding sequences with low-quality indices (used in Matranga et al. 2014).

A) Using the second approach, rodent contaminations was detected in three human samples. Each bar represents one sample, and the numbers (white color) in each bar indicate the number of rodent-specific miRNA sequences (bars below).

B) The samples demultiplexed using the two approaches have comparable amount of total reads.

C) The samples demultiplexed using the first approach contain more miRNA reads compared to the second approach.

D) The samples demultiplexed using the first approach have higher miRNA complexity.

More information about the analysis can be found in Supplementary Method section "Computational removal of mis-assigned reads based on Matranga et al. 2014".

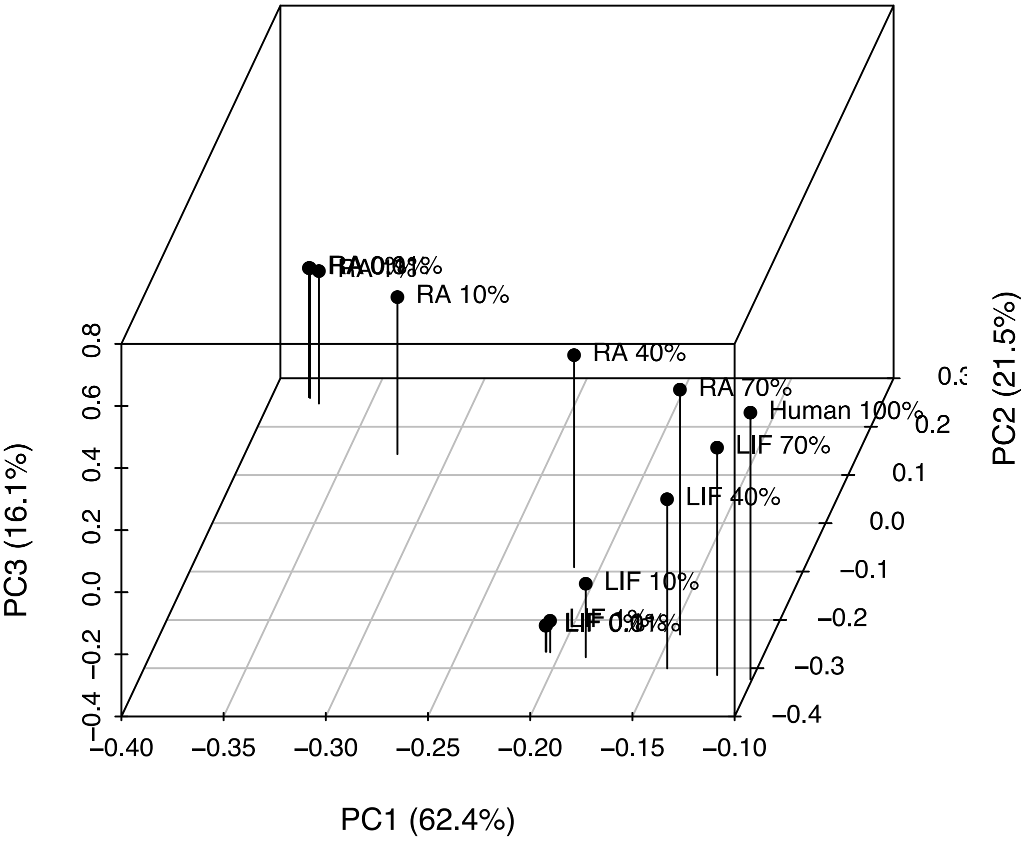

Figure S7: PCA of mouse samples with human contamination

The PCA plot shows how the miRNA profile changes with increasing the human contamination levels. The LIF and RA indicate mouse embryonic stem cells that had been cultivated in a medium to maintain their pluripotency (LIF) and a medium to stimulate differentiation to neuron (RA) respectively. The plot is the 3D version of main Figure 3F left panel.

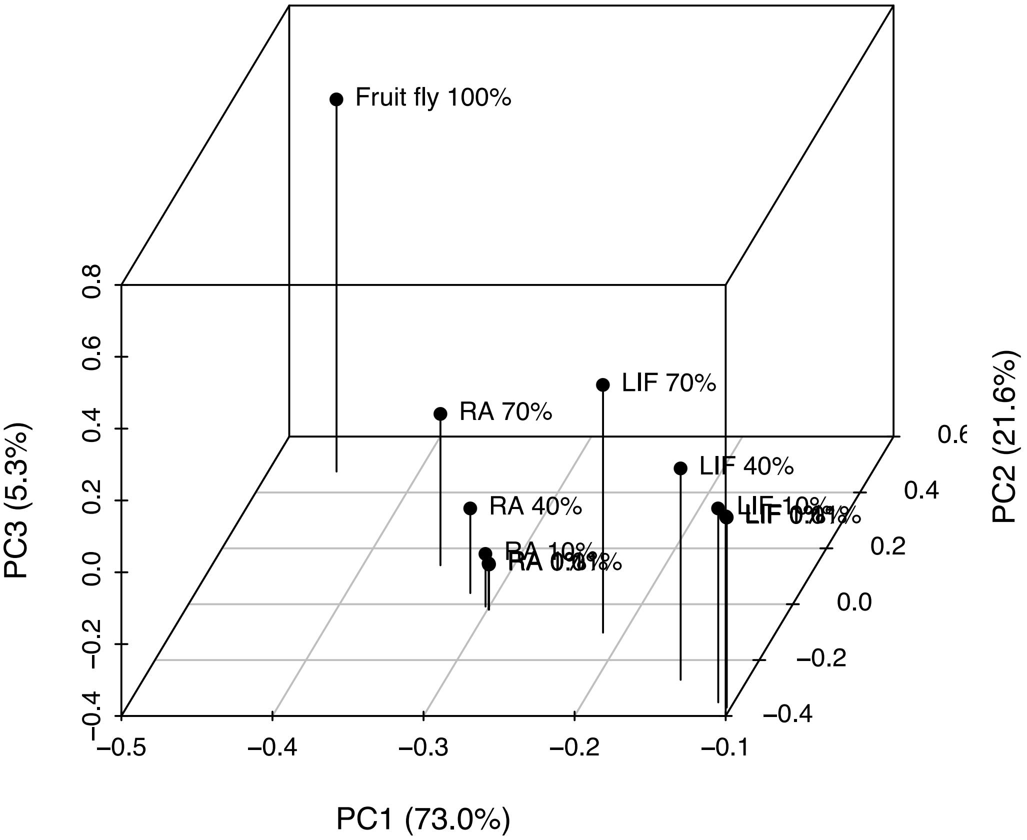

Figure S8: PCA of mouse samples with fruit fly contamination

The PCA plot shows how the miRNA profile changes with increasing the fly contamination levels. Abbreviations are the same as in Figure S7. The plot is the 3D version of main Figure 3F right panel.

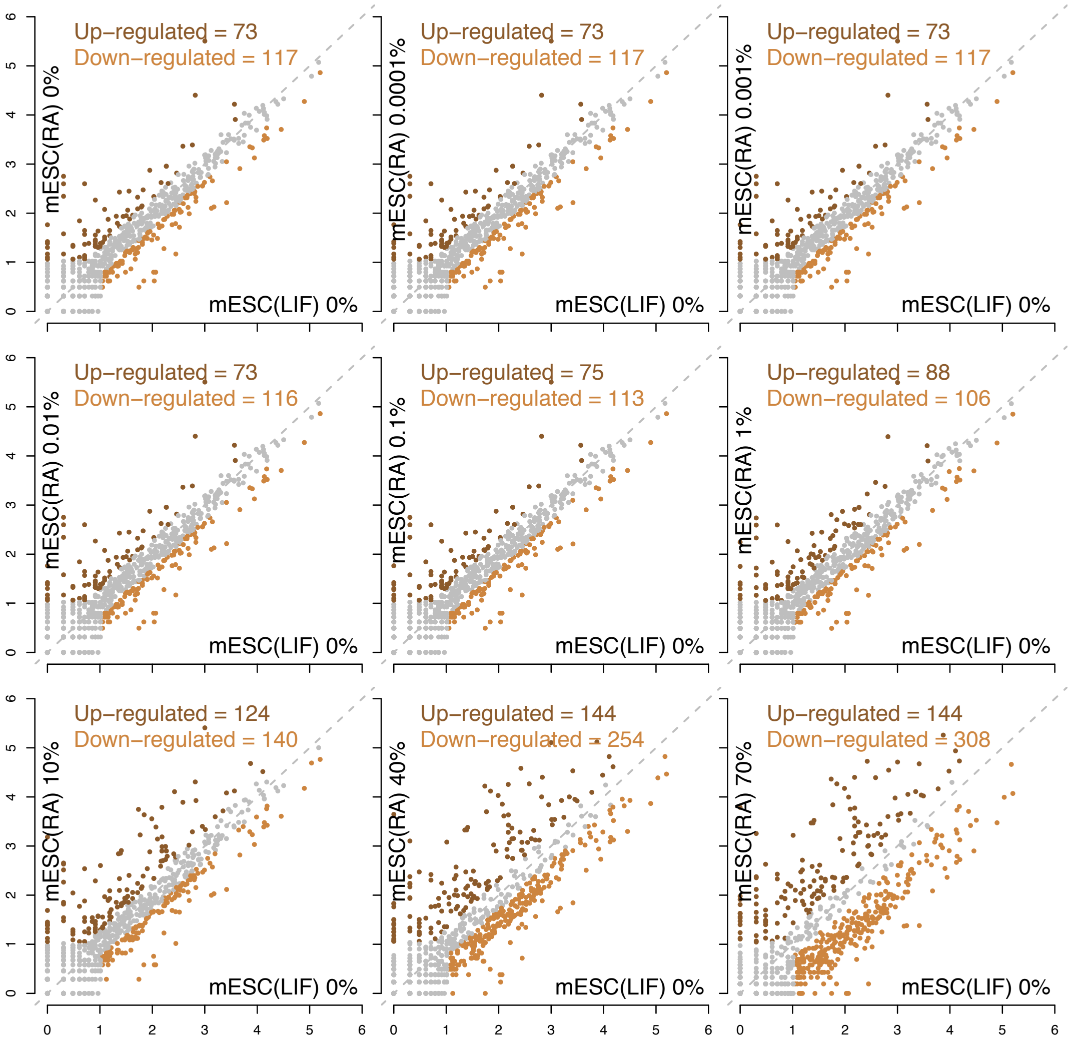

Figure S9: miRNA differential expression analysis of mouse samples with human contamination

The scatter plots show how many differentially expressed miRNAs are identified with increasing the contamination levels. The miRNA expression levels are indicated by log10 (RPM + 1) in x and y axis. The criteria to identify differentially expressed miRNAs: i) for miRNA expressed in both samples, requires ≥ 10 RPM in one of the samples and > 2 fold change in RPM expression between the two samples; ii) for miRNA expressed only in one sample, requires RPM ≥ 10.

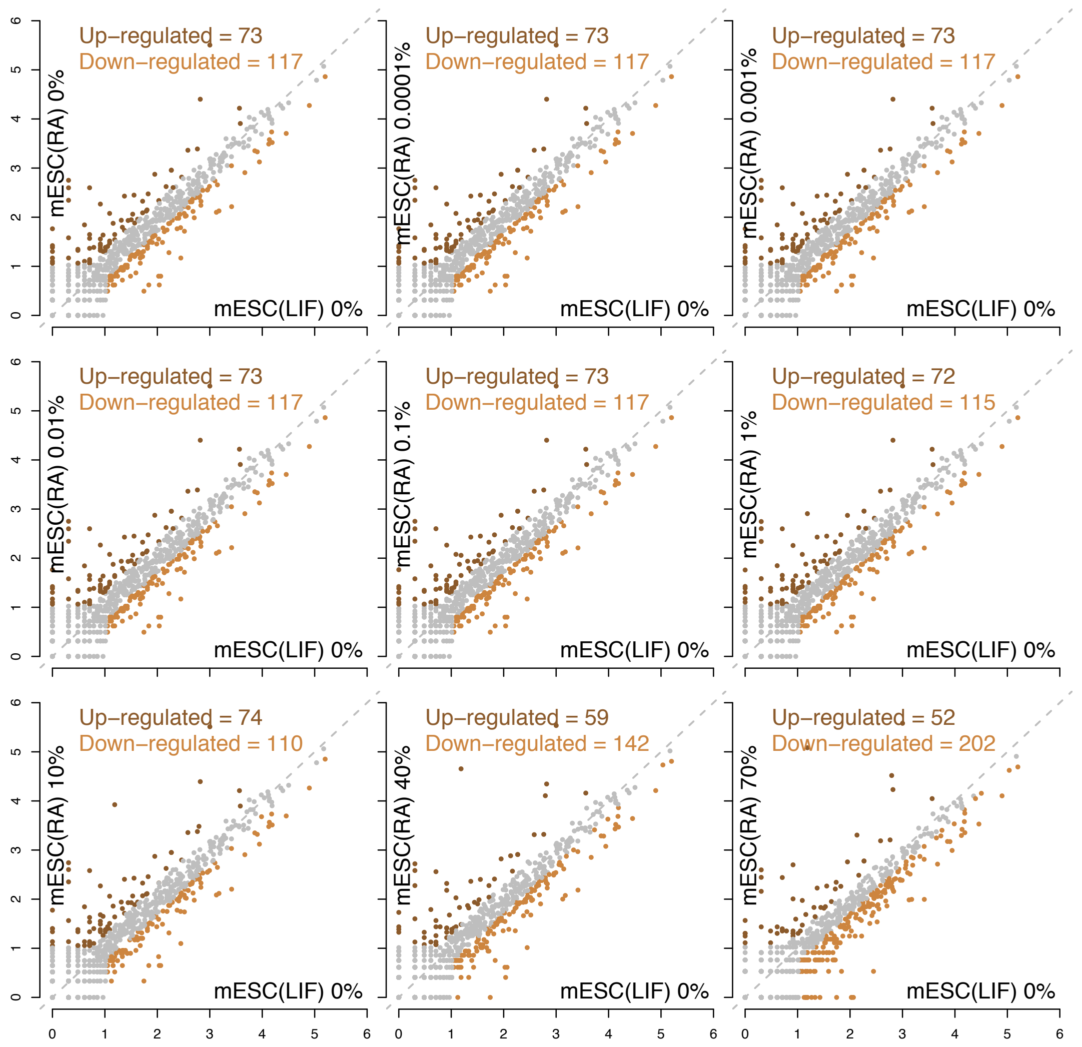

Figure S10: miRNA differential expression analysis of mouse samples with fruit fly contamination

The scatter plots show how many differentially expressed miRNAs are identified with increasing the contamination levels. The criteria are the same as in Figure S9.

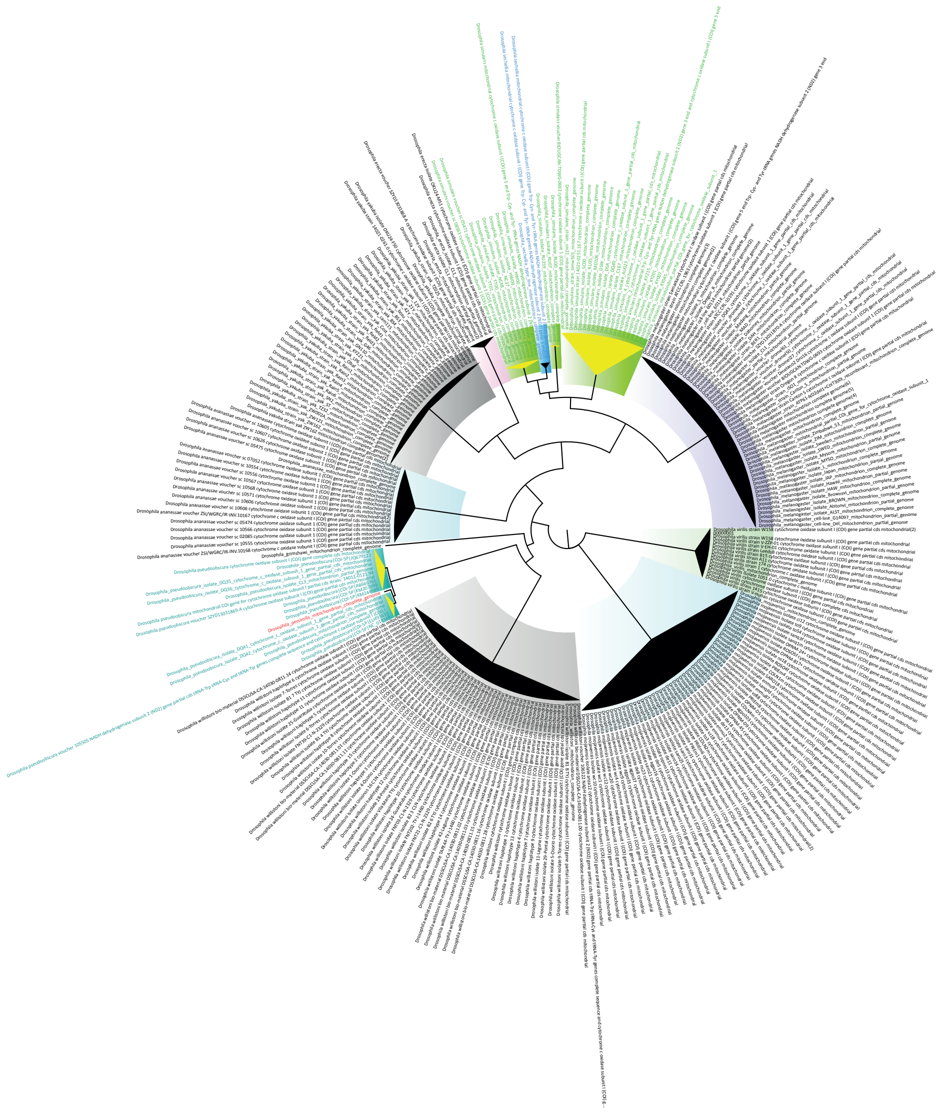

Figure S11: Neighbor-joining tree of COI sequences of the 12 *Drosophila* species

Neighbor-joining tree of aligned segments of approximately 600 base pairs of the mitochondrial gene cytochrome oxidase I (COI) ‘barcode segments’ for all available sequences for 12 *Drosophilid* species collected from NCBI (accession date: 31st of August 2018). While the majority of representatives clustered according to their species annotations in monophyletic groups (black triangles; 8 blocks that contain all representatives of exact one species: *D. willistoni*, *D. mojavensis*, *D. virilis*, *D. melanogaster*, *D. sechellia*, *D.erecta*, *D. yakuba*, *D. ananassae*), 2 described species were not resolved as a monophyletic group but were paraphyletic with respect to another species (yellow triangles; highlighted in petrol colored text: *D. pseudoobscura* included *D. persimilis* (red text), and highlighted in green text: *D. simulans* included monophyletic *D. sechellia* (blue text)). In these two cases the intra-species variations are larger than the inter-species variations. No statement about the monophyly or paraphyly of *D. grimshawi* or *D. persimilis* can be made as these species are only represented by one sample sequence.

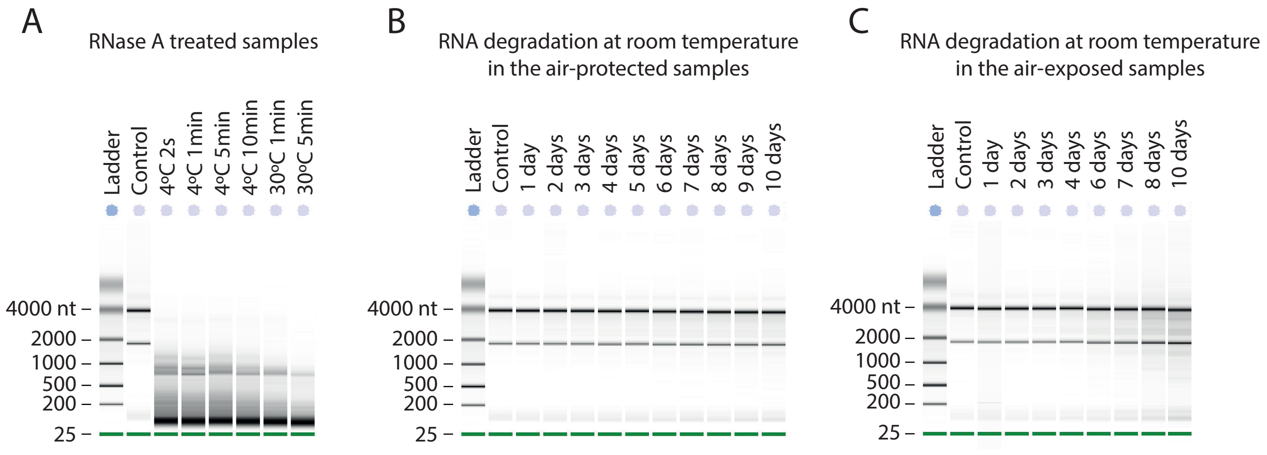

Sample Name	RIN
Control	10
4ºC 2s	2.30
4ºC 1min	2.30
4ºC 5min	2.20
4ºC 10min	2.30
30ºC 1min	2.30
30ºC 5min	2.40

Sample Name	RIN
Control	10
1 day	10
2 days	10
3 days	9.90
4 days	9.90
5 days	9.90
6 days	9.80
7 days	10
8 days	9.70
9 days	9.70
10 days	9.70

Sample Name	RIN
Control	10
1 day	10
2 days	10
3 days	10
4 days	10
6 days	9.70
7 days	9.20
8 days	8.70
10 days	8.20

Figure S12: RNA integrity assessment by RIN

A) Total RNA degradation at +4°C and +30°C in the RNase A treated samples.

B) Total RNA degradation at room temperature in the air-protected and air-exposed samples. No significant degradation of RNA was observed in the samples protected from air. In contrast, RNA exposed to air showed a slight degradation of 18S/28S rRNA. The RIN values dropped from 10.00 to 8.20 after 10 days at room temperature (C). More information about the experiment can be found in Supplementary Method section “RNA degradation at room temperature”.

References

1. Jung M, Schaefer A, Steiner I, Kempkensteffen C, Stephan C, Erbersdobler A, Jung K: **Robust microRNA stability in degraded RNA preparations from human tissue and cell samples.** *Clin Chem* 2010, **56:**998-1006.
2. Bravo V, Rosero S, Ricordi C, Pastori RL: **Instability of miRNA and cDNAs derivatives in RNA preparations.** *Biochem Biophys Res Commun* 2007, **353:**1052-1055.
3. Mathay C, Yan W, Chuaqui R, Skubitz AP, Jeon JP, Fall N, Betsou F, Barnes M, Group IBSW: **Short-term stability study of RNA at room temperature.** *Biopreserv Biobank* 2012, **10:**532-542.
4. Matranga CB, Andersen KG, Winnicki S, Busby M, Gladden AD, Tewhey R, Stremlau M, Berlin A, Gire SK, England E, et al: **Enhanced methods for unbiased deep sequencing of Lassa and Ebola RNA viruses from clinical and biological samples.** *Genome Biol* 2014, **15:**519.
5. Hebert PD, Cywinska A, Ball SL, deWaard JR: **Biological identifications through DNA barcodes.** *Proc Biol Sci* 2003, **270:**313-321.
6. Holtgrewe M: **Mason–a read simulator for second generation sequencing data.** *Technical Report FU Berlin* 2010.
7. Bioinformatics B: **FastQ Screen.** *Babraham Bioinformatics* 2013.
