# Supplementary material for: miRTrace reveals the organismal origins of microRNA sequencing data
Source: Genome Biol. 2018 Dec 4;19:213. doi: 10.1186/s13059-018-1588-9 (PMC6280396; doi:10.1186/s13059-018-1588-9)
Supplement: Supplementary file 4 — Supplementary miRTrace reports. Report S1. miRTrace Trace report of the samples used in Fig. 2. Report S2. miRTrace QC report of the public M. musculus small RNA-Seq datasets used in Fig. 3a. Report S3. miRTrace QC report of the public C. elegans small RNA-Seq datasets used in Fig. 3a. Report S4. miRTrace QC report of the public D. melanogaster small RNA-Seq datasets used in Fig. 3a. Report S5. miRTrace QC report of the mouse samples in silico contaminated with various amounts of human sequences. The same samples as in main Fig. 3f left panel. Report S6. miRTrace QC report of the mouse samples in silico contaminated with various amounts of fruit fly sequences. The same samples as in main Fig. 3f right panel. Report S7–S8. miRTrace QC report of the samples used in Additional file 2: Figure S4. (ZIP 2285 kb) [file 13059_2018_1588_MOESM4_ESM.zip › Additional file 4/Report S1.html]

PHRED Score Distribution

Percentage of nucleotides with given PHRED score.

Read Length Distribution

Percentage of reads of each length.

Quality Control Statistics

Percentage of reads with given quality control status.

RNA Type

Percentage of reads of each RNA type.

miRNA Complexity

Number of detected distinct miRNA genes as function of read depth.

Contamination

Percentage of clade-specific miRNA-reads belonging to each clade.

Sample Statistics

| Friedländer Lab
