# Supplementary material for: miRTrace reveals the organismal origins of microRNA sequencing data
Source: Genome Biol. 2018 Dec 4;19:213. doi: 10.1186/s13059-018-1588-9 (PMC6280396; doi:10.1186/s13059-018-1588-9)
Supplement: Supplementary file 4 — Supplementary miRTrace reports. Report S1. miRTrace Trace report of the samples used in Fig. 2. Report S2. miRTrace QC report of the public M. musculus small RNA-Seq datasets used in Fig. 3a. Report S3. miRTrace QC report of the public C. elegans small RNA-Seq datasets used in Fig. 3a. Report S4. miRTrace QC report of the public D. melanogaster small RNA-Seq datasets used in Fig. 3a. Report S5. miRTrace QC report of the mouse samples in silico contaminated with various amounts of human sequences. The same samples as in main Fig. 3f left panel. Report S6. miRTrace QC report of the mouse samples in silico contaminated with various amounts of fruit fly sequences. The same samples as in main Fig. 3f right panel. Report S7–S8. miRTrace QC report of the samples used in Additional file 2: Figure S4. (ZIP 2285 kb) [file 13059_2018_1588_MOESM4_ESM.zip › Additional file 4/Report S7.html]

PHRED Score Distribution

Percentage of nucleotides with given PHRED score.

Read Length Distribution

Percentage of reads of each length.

Quality Control Statistics

Percentage of reads with given quality control status.

RNA Type

Percentage of reads of each RNA type.

miRNA Complexity

Number of detected distinct miRNA genes as function of read depth.

Contamination

Percentage of clade-specific miRNA-reads belonging to each clade.

Sample Statistics

Usage Advice

Note: the **counts on top of each sample bar** show the number of reads used as input for this module.

Sample filtering tip: **samples can be selected by left-clicking** the sample bar (use the ctrl/command key to select multiple).

Sample filtering tip: selecting one or more samples will update the legends to show statistics for only these samples.

Navigation tip: to **quickly navigate** between the report types **use the W and S keys**.

Navigation tip: when one or more samples are selected (see above), one can **shift the current selection** using the **A or D keys**.

Layout tip: when using the **"Compress reports" button** in the top bar, the page switches to a compact representation.

VIM tip: users familiar with the VIM editor can use the HJKL keys instead of WASD.

For more information, see the PDF manual or go to the miRTrace web site.

| Friedländer Lab
