# Supplementary material for: miRTrace reveals the organismal origins of microRNA sequencing data
Source: Genome Biol. 2018 Dec 4;19:213. doi: 10.1186/s13059-018-1588-9 (PMC6280396; doi:10.1186/s13059-018-1588-9)
Supplement: Supplementary file 5 — Review history. (DOCX 281 kb) [file 13059_2018_1588_MOESM5_ESM.docx]

**First round of review**

**Reviewer 1**

**Are the methods appropriate to the aims of the study, are they well described, and are necessary controls included?**
Generally yes, see comments below.

**Are the conclusions adequately supported by the data shown?**

Generally yes, see comments below.

**Are sufficient details provided to allow replication and comparison with related analyses that may have been performed?**

Generally yes, see comments below.

**Does the method perform better than existing methods (as demonstrated by direct comparison with available methods)?**

No: Not sure if there are existing methods for miRNAs, but there are other methods for rRNA or specific genes. See below.

**Is the method likely to be of broad utility? Is any software component easy to install and use?**

Broad utility – probably limited to areas such as forensic science, clinical parasitology, and food quality control. Cannot comment on software installation or use.

**Is the paper of broad interest to others in the field, or of outstanding interest to a broad audience of biologists?**

No.

**Comments to authors**

This manuscript describes a new computational method, miRTrace, which can use miRNA sequence data to assign reads to their taxonomic origins. The authors suggest that this method will have applications in the fields of forensic science, clinical parasitology, and food quality control. [Note that this reviewer has no expertise in any of those fields.] Previous literature, such as reference 7, clearly support the use of miRNAs for this purpose. It is less clear that there is an advantage to their approach over the use of ribosomal RNA (rRNA) or other genes - their approach requires the generation of short RNA sequence data, which is probably less widely available than other RNA-Seq datasets. Their actual method seems to work well based on the results shown in this manuscript and my primary concern with this manuscript is its organization. The authors' computational solution to find cross-contaminated Illumina reads is clever and an interesting advance.

The manuscript would be easier to follow if it was presented in a reorganized manner. In particular, miRTrace should be explained at the beginning of the Results section before any data are presented. Supplementary Figure 1 should be part of the main text. Figures 1A-D could be moved to the Supplementary material. It is worth adding information about why the analysis pipeline was designed as it was. In addition, the first sentence of the Results refers to 4 figures and 2 tables, but does not provide sufficient explanation of what is reported there and why they are included. 

The section entitled "miRTrace quality control accurately identifies problematic samples" would be better at the beginning of the manuscript because it explains the format of the Reports. For a reader, seeing all these Reports without the necessary background, which is presented in this section towards the end of the Results section, is confusing and hard to follow. Even with the information provided in that section, it's not always clear from a Report which are the key points that the authors want to emphasize for a particular conclusion.

The following concern is also worthy of additional attention. How does miRTrace compare to methods that use other genes to identify the origin of reads? The authors state the theoretical advantages of their method in the Introduction, but there are no data to support the use of this approach over others that presumably are in use already. Moreover the authors state that their method "requires no expert knowledge" and that should be modified to explain the extent to which other methods require such knowledge and theirs does not.


Minor points.
1) Page 2, line 31: Not sure how common this "common laboratory challenge" is.
2) Page 3, line 63: The authors should explain the term "barcoding gene analysis."
3) Page 6, line 144: It's hard to critically interpret Supplementary Figure 4 with the project labels at the top because the details of the experiments are not known. For example, could the plant RNA be from food sources for the mice?
4) Page 6, line 148: Do the authors see a difference among Illumina sequencing platforms for cross-contamination? The "index hopping" phenomenon has been reported to vary depending on the sequencing platform.
5) Page 7, line 169: Did the authors consider discarding index reads with lower quality bases as was done in Matranga et al. Genome Biology (2014) 15:519?
6) Page 7, line 178: It would be helpful to show details about how long the reads need to be to use this approach to read the index read in the non-index read.
7) Page 8, line 210 & Page 11, line 281: It would be better to refer to these datasets as "potentially compromised by contaminants" as the extent of the problems in each dataset is not demonstrated directly.
8) Page 8, line 215 & Page 14, line 355: The term "user-friendly" is subjective and should be used only in the Discussion or other areas of the manuscript that are places for opinions.
9) Page 16, line 424: Is Supplementary Figure 12 missing? It was not clear where to find it.
10) Page 17, line 433: The authors should provide more information on how the libraries were constructed. While information on the Illumina TruSeq small RNA kit v2 may be available now, it would be better to have some additional information presented, such as a reference and a brief description, so that readers in the future can know how the experiments were done.
11) Figure 1F: Is it possible to use such a small number of reads and still have confidence in the results?
12) Figure 2B: Would it be possible to add information on what parameters the box plot is showing (gray area, dark line, white dot)?
13) Figure 3: Are the annotations to the right of panels B, C, and D part of the report or just added for the manuscript?
14) Supp. Info. Page 4: What is a "single barcoding gene locus"?
15) Supp. Info. Page 4: RNA degradation likely depends on other variables besides temperature and time - such as contaminating traces of RNases, divalent cations, and other destabilizing elements. It's not possible to capture all these variables in an experiment with a single sample. 
16) Supp. Info. Page 4: The authors should rephrase the final sentence "… does not decrease significantly RNA quality" to reflect the fact that RIN score is an assessment of ribosomal RNA integrity, but not small RNA or mRNA integrity.
17) Supp. Info. Page 5: Does this calculation take into account the base composition (GC content) of the samples?
18) Supp. Figure 6: The "Heatmap showing the indices of contamination reads" seems hard to read - is it gray scale? No scale is shown for the heatmap. And the word "gridence" does not seem to be an English word. Overall, this is a confusing figure.

**Recommended: Major revisions required.**

**Reviewer 2**

**Are the methods appropriate to the aims of the study, are they well described, and are necessary controls included?**
Yes on all points. The controls are especially appreciated, checking titrations of cross-contaminating samples, incorrect adapters, and partial degradation.

**Are the conclusions adequately supported by the data shown?**

Yes, the data clearly support the utility of the miRTrace program in assigning detected miRNAs to organism (or group) of origin.

**Are sufficient details provided to allow replication and comparison with related analyses that may have been performed?**

Yes. The program is currently available with a password and will presumably be made available publicly after publication.

**Does the method perform better than existing methods (as demonstrated by direct comparison with available methods)?**

Yes.

**Is the method likely to be of broad utility? Is any software component easy to install and use?**

Yes, it will be used broadly. The software can be used by anyone with basic computing skills and knowledge of sequencing data.

**Is the paper of broad interest to others in the field, or of outstanding interest to a broad audience of biologists?**

Yes. It will be of broad interest to miRNA and biomarker researchers.

**Comments to authors**

1. I wonder how the method would perform for library preparation procedures that are not biased towards miRNA, for example CATS or Clontech unbiased preps? Data would be interesting, but discussion of this might suffice.
2. The data suggest substantial presence of plant miRNAs in animal samples. Does this support the so-called “dietary xenomiR” hypothesis the authors covered in ref 31?
3. A minor point, but a careful read/revision of the text is needed to solidify the language used. For example, “contamination” is not always the right word. The miRTrace program cannot distinguish between all of the different possible ways in which unexpected miRNA was introduced. For example, in the parasitic infection example, parasitic miRNAs are detected, but does this mean the sample was contaminated, or the animal was infected? ON the other hand, the program may identify problems due to degradation.

**Recommended: Minor revisions required.**

**Authors’ response to reviewers**

**Reviewer 1 comments (italics) and response from authors**

*This manuscript describes a new computational method, miRTrace, which can use miRNA sequence data to assign reads to their taxonomic origins. The authors suggest that this method will have applications in the fields of forensic science, clinical parasitology, and food quality control. [Note that this reviewer has no expertise in any of those fields.] Previous literature, such as reference 7, clearly support the use of miRNAs for this purpose. It is less clear that there is an advantage to their approach over the use of ribosomal RNA (rRNA) or other genes - their approach requires the generation of short RNA sequence data, which is probably less widely available than other RNA-Seq datasets. Their actual method seems to work well based on the results shown in this manuscript and my primary concern with this manuscript is its organization. The authors' computational solution to find cross-contaminated Illumina reads is clever and an interesting advance.*

Response: we thank the referee for their interest in our study and for their positive comments. We address his concerns regarding comparison to other tracing methods and the need for reorganizing the manuscript below.

*The manuscript would be easier to follow if it was presented in a reorganized manner. In particular, miRTrace should be explained at the beginning of the Results section before any data are presented. Supplementary Figure 1 should be part of the main text. Figures 1A-D could be moved to the Supplementary material. It is worth adding information about why the analysis pipeline was designed as it was. In addition, the first sentence of the Results refers to 4 figures and 2 tables, but does not provide sufficient explanation of what is reported there and why they are included. The section entitled "miRTrace quality control accurately identifies problematic samples" would be better at the beginning of the manuscript because it explains the format of the Reports. For a reader, seeing all these Reports without the necessary background, which is presented in this section towards the end of the Results section, is confusing and hard to follow. Even with the information provided in that section, it's not always clear from a Report which are the key points that the authors want to emphasize for a particular conclusion.*

Response: we thank the referee for this suggestion. We have now substantially reorganized the manuscript, introducing two new figures, and re-writing the Introduction, Results and Discussion sections. All changes are highlighted in the manuscript in red fonts. Specifically, we have introduced two new main figures, Figure 1 and Figure 4, that show the work-flow of miRTrace in its *Trace* mode and in its *QC* mode respectively. We have also substantially expanded the figure with the *Drosophila* case study (current Figure 6) to include comparison with two other commonly used tracing methods, as requested. The Results section has been substantially expanded to reflect these new figures and analyses, and we have included more text in the Introduction to better explain the concept of gene barcoding. Last, we have added new text to the Discussion section to add more substance, since the *Drosophila* case study was moved from this section to the Results. As mentioned, in the new Figure 1 we focus on the *Trace*

functionality, since we think this is the unique feature of miRTrace. We still retain the *QC* aspects, along with the extensive full-page report, in the later section. While the full-page report is important, we agree with the referee that it is complicated. Therefore, we think that the simplified and focused flow-chart and example output in Figure 1 is a better introduction to our new method. As proposed, we now also start the Results section text with a detailed explanation of the pipeline. We think that the reorganization and the new figures and text part have improved our manuscript, and we hope that the referee agrees.

*Comment: the following concern is also worthy of additional attention. How does miRTrace compare to methods that use other genes to identify the origin of reads? The authors state the theoretical advantages of their method in the Introduction, but there are no data to support the use of this approach over others that presumably are in use already.*

Response: first, it is important to emphasize that miRTrace currently is the only existing method to trace miRNA sequencing data back to their taxonomical origins. Therefore, it is not possible to make a comparison to tools with the same functionality; in its field miRTrace is unique. However, we agree with the referee that it is interesting and important to compare miRTrace to similar methods that are applied in related fields. This is both interesting as an interdisciplinary comparison, but also to gauge if miRNAs are generally promising markers for tracing taxonomy, compared to other markers. We have therefore performed two major comparisons, using the same 12 *Drosophila* species for all analyses. To our knowledge, we have chosen the two most widely used tracing methods for our comparisons (Barcode of Life and FastQ Screen).

In the first analysis, we compared miRTrace to the Barcode of Life Data Systems Identification Engine. This is in essence a blast service, where users can search their COI sequence of interest against all COI sequences in the Barcode of Life database, which includes sequences from ~200,000 species. We here simulated Sanger sequences for the twelve Drosophila species, one thousand sequences for each species, considering intra-species variation and common Sanger sequencing error rates. We found that the Barcode of Life Engine assigned more sequences correctly than miRTrace for seven of the twelve species, while miRTrace performed better for four species, and both methods performed perfectly on one species (Figure 6A-B). Importantly, the Barcode of Life Engine was unable to unambiguously assign reads for two species, likely because of sequence mis-annotations in their database.

In the second analyses, we compared miRTrace to FastQ Screen, which assigns RNA sequencing reads to species using whole-genome mapping. This tool is commonly used to detect contamination in sequencing data. In our comparison, miRTrace outperformed FastQ Screen for five out of twelve species, while FastQ Screen performed better for seven species (Figure 6A and Figure 6C). It is important to note that these *Drosophila* species comprise difficult use cases for FastQ Screen, since some species are closely related and several genomes are incompletely assembled.

In summary, it appears that miRTrace is comparable in performance to tracing methods that are state-of-the-art in their respective fields. We think this is impressive given that miRNAs are hort sequences (~22 nucleotides) compared to the COI gene (~600 nucleotides) or the typical RNA sequencing read (~100 nucleotides). This short length makes it more challenging to assign reads unambiguously. But as we mention in the updated Discussion section, this also affects the efficiency of the respective tracing methods – it is of course much faster to look for perfect matches to a catalog of ~22 nucleotide sequences representing animal and plant clades than it is to search for matches in multiple large genomes. It is still too early to say if miRNAs can compete with e.g. barcoding genes for a general role in the tracing field. But the results of our comparisons here do not rule out such a role. Importantly, miRTrace is currently the only method to trace miRNA sequencing data to their taxonomic origins, and we foresee that this in itself will be immensely beneficial to the community.

*Comment: moreover the authors state that their method "requires no expert knowledge" and that should be modified to explain the extent to which other methods require such knowledge and theirs does not.*

Response: we initially wrote this sentence referring to rDNA analyses, which in some cases require construction of phylogenetic trees from sequence similarity. However, since we in the rest of our study compare our method with COI barcoding analyses, which are more relevant for eukaryotes and are also simpler to perform, we have removed our statement on expert knowledge from the manuscript.

*Comment: minor points. 1) Page 2, line 31: Not sure how common this "common laboratory challenge" is.*

Response: we agree, and we have changed the phrasing to "important laboratory challenge". This is from the point of view that applications to forensics and food quality control are important, as are detecting laboratory contaminations when they are present.

*Comment: 2) Page 3, line 63: The authors should explain the term "barcoding gene analysis."*

Response: we have now included a new section in the Introduction, to explain the concept of barcoding gene analysis. In addition to this specific section, we have generally included more detail and background in the Introduction, along expanded and updated references.

*Comment: 3) Page 6, line 144: It's hard to critically interpret Supplementary Figure 4 with the project labels at the top because the details of the experiments are not known. For example, could the plant RNA be from food sources for the mice?*

Response: there is compelling evidence from recent studies that these "xenomiRs" are contamination rather than dietary. We discuss this in a recent letter to the Journal of Nutritional Biochemistry (www.sciencedirect.com/science/article/pii/S0955286318305357). However, we actually do not want to engage this topic in the current study, where we conservatively focus on the presence of primate sequences in data from mouse, nematode and fly (Figure 2A-B in the updated manuscript). In these cases, the most likely explanation is researcher contamination - in any case it is inconceivable how primate miRNAs would end up in a mouse sample for biological or dietary reasons. We agree that it was distracting that we previously included plant sequences in the Supplementary Figure 4; we have now omitted these and only show the presence of primate sequences, which almost certainly originate from *bona fide* contamination.

*Comment: 4) Page 6, line 148: Do the authors see a difference among Illumina sequencing platforms for cross-contamination? The "index hopping" phenomenon has been reported to vary depending on the sequencing platform.*

Response: our samples were sequenced on the Illumina NextSeq500 instrument. The index misreading and mis-paring events observed in our study are distinct types of errors from index hopping. Index hopping is more common on newer Illumina machines such as HiSeq 3000/4000/X Ten, which use exclusion amplification chemistry on patterned flow cells to generate clusters. In contrast, index misreading and mis-pairing events are more general and are not associated with particular Illumina sequencing platforms. For example, index misreading can result from multiple sources of errors in PCR amplification, sequencing and so on. Index mis-pairing could be linked to mixed clusters that can be formed if colonies from two different template molecules grow into each other during cluster generation (Kircher et al., Nucleic Acids Research, 2012). In summary, the "index hopping" phenomenon would add yet a level of complexity to the technical challenges that we describe, and we feel that this is beyond the scope of our present study.

*Comment: 5) Page 7, line 169: Did the authors consider discarding index reads with lower quality bases as was done in Matranga et al. Genome Biology (2014) 15:519?*

Response: we have made a comparison between our cleaning method and that of Matranga et al., Genome Biology (2014) (new Supplementary Figure 6). We find that both methods are equally efficient in removing contaminating reads (Suppl. Fig. 6A). However, our method retains more of the useful miRNA reads originating from slightly more distinct miRNA genes (Suppl. Fig. 6C-D). Importantly, when sequence data are deposited to public repositories such as SRA, the index quality scores are almost always omitted. This means that the Matranga et al. method can practically be used only on in-house data, while our method can be used on both in-house and public data.

*Comment: 6) Page 7, line 178: It would be helpful to show details about how long the reads need to be to use this approach to read the index read in the non-index read.*

Response: in order to apply our approach (Figure 3E) to remove reads with inconsistent indices, the sequencing reads should be long enough to cover the index sequences. According to the structure of Illumina read: miRNA (~22 nt) – 3’ adapter (33 nt) – index (6 nt), the read should be at least 61 nucleotides long. This means that the default 75bp Illumina small RNA TruSeq kit works, while sequencing only 50 nucleotides would not work.

*Comment: 7) Page 8, line 210 & Page 11, line 281: It would be better to refer to these datasets as "potentially compromised by contaminants" as the extent of the problems in each dataset is not demonstrated directly.*

Response: we have gone through the manuscript carefully and evaluated every instance of the word "contaminants", changing it where necessary. In particular, we have in several places changed "contamination" to "evidence of contamination". As mentioned above, we conservatively limit our analyses to the presence of primate sequences, but we agree that it is better to use a cautious wording.

*Comment: 8) Page 8, line 215 & Page 14, line 355: The term "user-friendly" is subjective and should*

*be used only in the Discussion or other areas of the manuscript that are places for opinions.*

Response: we agree and have replaced the term "user-friendly" in these instances with "portable". This adjective seems reasonable, since Java source can run on a myriad of devices.

*Comment: 9) Page 16, line 424: Is Supplementary Figure 12 missing? It was not clear where to find it.*

Response: we thank the referee for pointing this out. This was a mistake and we have made sure that this is corrected (incidentally there now is a Supplementary Figure 12, since we have added more figures to the supplementary materials).

*Comment: 10) Page 17, line 433: The authors should provide more information on how the libraries were constructed. While information on the Illumina TruSeq small RNA kit v2 may be available now, it would be better to have some additional information presented, such as a reference and a brief description, so that readers in the future can know how the experiments were done.*

Response: we agree, and we have included a more extensive description of the protocol used in the Methods section.

*Comment: 11) Figure 1F: Is it possible to use such a small number of reads and still have confidence in the results?*

Response: in comparison, traditional barcoding typically relies on a single sequence readout, obtained by Sanger sequencing, to perform taxonomical tracing. It is also worth noting that the data that we present in this figure are from single cells, so the marker molecules are limited in number for biological reasons. The current methods to sequence miRNAs in single cells are inefficient (around one miRNA read per 2000 total reads), however, miRTrace will provide uch more reliable results once more efficient single cell miRNA sequencing protocols are developed. We consider this subfigure to be a proof-of-principle.

*Comment: 12) Figure 2B: Would it be possible to add information on what parameters the box plot is showing (gray area, dark line, white dot)?*

Response: we have included this information in the figure legend.

*Comment: 13) Figure 3: Are the annotations to the right of panels B, C, and D part of the report or just added for the manuscript?*

Response: we have added these annotations for the manuscript. In order to make the report easier to understand, the extra arrows and labels were added to the figure of the miRTrace report. We have clarified this in the figure legend.

*Comment: 14) Supp. Info. Page 4: What is a "single barcoding gene locus"?*

Response: a single barcoding gene locus could for instance be the COI locus. We here use the term to discern methods that rely on the information from a single gene, as traditional barcoding methods do, from the methods that rely on information from multiple genes, as our method does.

*Comment: 15) Supp. Info. Page 4: RNA degradation likely depends on other variables besides temperature and time - such as contaminating traces of RNases, divalent cations, and other destabilizing elements. It's not possible to capture all these variables in an experiment with a single sample.*

*Comment: 16) Supp. Info. Page 4: The authors should rephrase the final sentence "… does not decrease significantly RNA quality" to reflect the fact that RIN score is an assessment of ribosomal RNA integrity, but not small RNA or mRNA integrity.*

Response: we agree with the two comments, and we have changed the sentence ‘Therefore, short-term storage of total RNA samples at room temperature does not decrease significantly RNA quality’ to ‘This suggests that short-term storage of total RNA samples at room temperature does not necessarily cause a substantial decrease of RNA quality, as measured by rRNA integrity’.

*Comment: 17) Supp. Info. Page 5: Does this calculation take into account the base composition (GC content) of the samples?*

Response: this calculation uses the simplifying assumption that nucleotides in the genome are identically and independently distributed, in other words that each of the four nucleotides occur with 25% frequency. The message of this supplementary figure is that false positives caused by random sequence (e.g. technical noise of sequencing instruments) will be arbitrarily rare – less than one false positive read in a typical 10 million read experiment.

*Comment: 18) Supp. Figure 6: The "Heatmap showing the indices of contamination reads" seems hard to read - is it gray scale? No scale is shown for the heatmap. And the word "gridence" does not seem to be an English word. Overall, this is a confusing figure.*

Response: we agree that this figure is quite complicated. We have simplified the figure and rewritten the figure legend text so that it is hopefully easier to understand.

**Reviewer 2 comments (italics) and response from authors**

*Comment: 1. I wonder how the method would perform for library preparation procedures that are not biased towards miRNA, for example the CATS or Clontech unbiased preps? Data would be interesting, but discussion of this might suffice.*

Response: when we first started developing miRTrace, almost all small RNA studies were conducted using the Illumina TruSeq protocol. Now, several new promising protocols have emerged, and we feel that it is important that miRTrace can also handle data generated using these new protocols. Therefore, we have improved our software so it is now compatible with CATS, QiaSeq and NextFlex small RNA sequencing data, using the completely new command line option ‘--protocol’. These option means that the adapter trimming step is adapted for the adapter architecture of the specific protocol. We obtain good results from analyzing in-house and public data, as evidenced by the length distribution and RNA type plots shown on the next page. Specifically, the length profiles of the QiaSeq and NextFlex data peak at 22 nucleotides, as expected from high-quality miRNA sequencing data, and these data sets also has a high miRNA content. As expected, the CATS data is less biased towards miRNAs, but the smooth length profile shows that most adapters have been removed, and the data sets have a high rRNA content, which probably represents a large pool of degradation products. In summary, our updated software performs well on these new data types in our testing cases, but we will of course continue to improve it as we get more feedback from researchers using our method.

*Comment: 2. The data suggest substantial presence of plant miRNAs in animal samples. Does this support the so-called “dietary xenomiR” hypothesis the authors covered in ref 31?*

Response: this is an interesting question, which Referee 1 also asked. We have answered the question in comment 3 above. In brief, there is substantial evidence that xenomiRs are contamination rather than dietary, which we have discussed in a recent letter to the Journal of Nutritional Biochemistry ([www.sciencedirect.com/science/article/pii/S0955286318305357](http://www.sciencedirect.com/science/article/pii/S0955286318305357)). In the figure where we present putative contamination in public data (the new Figure 2A-B) we conservatively consider only on the presence of primate sequences in data from mouse, nematode and fly. In these cases, the most likely explanation is researcher contamination, or index mis-assignment of human sequences from e.g. patient studies.

*Comment: 3. A minor point, but a careful read/revision of the text is needed to solidify the language used. For example, “contamination” is not always the right word. The miRTrace program cannot distinguish between all of the different possible ways in which an unexpected miRNA was introduced. For example, in the parasitic infection example, parasitic miRNAs are detected, but does this mean the sample was contaminated, or the animal was infected? On the other hand, the program may identify problems due to degradation.*

Response: we completely agree, and we have gone carefully through the manuscript and evaluated every instance of “contamination”, changing it where necessary (see also our response to Comment 7 of Referee 1, above).

**QiaSeq**

**NextFlex**

**CATS**

**Second round of review**

Reviewer 1

The revised manuscript is much improved in two key ways. First, the organization and flow is much clearer in the revised version. Second, the authors have included comparisons to other methods for identifying the taxonomic origin of sequence reads. In relation to the comparison made, it seems that miRTrace is not substantially better in all regards, but has comparable performance and would be a worthy complementary approach to the existing tools. It is not clear whether the field will adopt the use of miRNAs for this purpose as they require specialized RNA isolation and library construction, which are not that difficult – just outside of standard practice for some scientists. 

Following up on more minor points. 
1) Comment 4 from initial review. Would it be possible for the authors to compare the level of cross contamination across the different sequencing platforms in the public data that they used? It is fine for them to report this information without performing any additional analysis. 

2) Comment 6 from initial review. The authors should mention somewhere in the manuscript that a read length of 61 nucleotides is required for this approach. It might seem obvious, but this is the type of detail that could escape notice until it was too late to use this clever approach. 

3) In the new section comparing miRTrace to the use of the COI gene in a simulation, there are two details missing about how the simulated Sanger reads were created. First, were they created using standard PCR primer pairs as the ends of the reads? It seems like this would follow the experimental detail provided (“PCR amplify the COI gene and sequence it with Sanger sequencing.”). Second, how long are the simulated Sanger reads? Do they extend through the entire COI gene? Enough detail should be reported for another scientist to repeat this analysis. 

4) Supplementary Figure 1B. Why include labels for data points if the text is not legible for many of them? 

5) Supplementary Figure 5. Should there be 24 rectangles for each sample? If not, what is the logic? Also, what do the light and dark blue and red colors signify, if anything?

**Authors’ response to reviewers**

**Reviewer 1 comments (italics) and response from authors**

*The revised manuscript is much improved in two key ways. First, the organization and flow is much clearer in the revised version. Second, the authors have included comparisons to other methods for identifying the taxonomic origin of sequence reads. In relation to the comparison made, it seems that miRTrace is not substantially better in all regards, but has comparable performance and would be a worthy complementary approach to the existing tools. It is not clear whether the field will adopt the use of miRNAs for this purpose as they require specialized RNA isolation and library construction, which are not that difficult – just outside of standard practice for some scientists.*

We thank the referee for the kind observations. We agree that the revision has really improved the manuscript and the study. It is true that it is not clear if miRNAs will be used for generalpurpose taxonomic tracing in the future - this will likely hinge on technological developments in miRNA profiling. However, we are confident that the use of miRTrace as a quality control software will prove invaluable to the miRNA community in both the short and the long perspective.

*1) Comment 4 from initial review. Would it be possible for the authors to compare the level of cross contamination across the different sequencing platforms in the public data that they used? It is fine*

*for them to report this information without performing any additional analysis.*

Response: we have carefully investigated which platforms the ~700 public data sets were generated from. Unfortunately, none of the data sets originated from platforms using the ExAmptechnology (e.g. HiSeq4000 or NovaSeq), so we cannot estimate the effect of ‘index hopping’. Most of the data sets (594 data sets) were generated on HiSeq instruments (version 2500 and older), while a substantial number (79 data sets) were generated on the old Illumina Genome Analyzer instruments (version I and II). We observe that the percentage of data sets contaminated with primate sequences is higher for the Genome Analyzer data (12/79 or 15%) compared to the HiSeq data (38/594 or 6%) using a binomial test (p-value < 0.01), but this does not change our overall conclusions.

*2) Comment 6 from initial review. The authors should mention somewhere in the manuscript that a*

*read length of 61 nucleotides is required for this approach. It might seem obvious, but this is the type*

*of detail that could escape notice until it was too late to use this clever approach.*

Response: we agree with the referee, and we have added this line to the relevant paragraph: ‘For instance, the default 75 bp Illumina TruSeq kit should be long enough to cover miRNA (~22 nt), adapter sequence (33 nt) and index (6 nt).’

*3) In the new section comparing miRTrace to the use of the COI gene in a simulation, there are two details missing about how the simulated Sanger reads were created. First, were they created using standard PCR primer pairs as the ends of the reads? It seems like this would follow the experimental detail provided (“PCR amplify the COI gene and sequence it with Sanger sequencing.”). Second, how long are the simulated Sanger reads? Do they extend through the entire COI gene? Enough detail should be reported for another scientist to repeat this analysis.*

Response: the simulation of the Sanger sequences did not consider PCR amplification. This makes our COI analyses more conservative, since PCR amplification could introduce additional errors that we do not consider. The simulated Sanger reads cover the 639 nucleotides sequence of the Genbank HM10229 sequence mentioned in the Supplementary Methods. In our opinion, this Methods paragraph is detailed enough for other researchers to repeat the analysis.

*4) Supplementary Figure 1B. Why include labels for data points if the text is not legible for many of them?*

Response: we agree, and we have reduced the size of the labels so that the overlap between them is minimized.

*5) Supplementary Figure 5. Should there be 24 rectangles for each sample? If not, what is the logic? Also, what do the light and dark blue and red colors signify, if anything?*

Response: we have clarified the text by elaborating this line: ‘Each sample can thus have 25 different indices: the expected (correct) index and 24 that result from sequencing errors at various positions. There are 24 possible indices assuming a single mismatch error, since the index is 6 nucleotides long, and a sequencing error can occur by any of these positions being converted into one of the three wrong nucleotides or an ‘N’, representing an uncalled nucleotide’. The light and dark shading of the rectangles do not have a significance, but they help discerning the rectangles from each other by visual inspection.
